# Breaking the barrier to biomolecule limit-of-detection via 3D printed multi-length-scale graphene-coated electrodes

**DOI:** 10.1038/s41467-021-27361-x

**Published:** 2021-12-06

**Authors:** Md. Azahar Ali, Chunshan Hu, Bin Yuan, Sanjida Jahan, Mohammad S. Saleh, Zhitao Guo, Andrew J. Gellman, Rahul Panat

**Affiliations:** 1 Department of Mechanical Engineering, Carnegie Mellon University, Pittsburgh, PA 15213 USA; 2 Department of Chemical Engineering, and Wilton E. Scott Institute for Energy Innovation, Carnegie Mellon University, Pittsburgh, PA 15213 USA

**Keywords:** Graphene, Biomedical engineering, Biosensors

## Abstract

Sensing of clinically relevant biomolecules such as neurotransmitters at low concentrations can enable an early detection and treatment of a range of diseases. Several nanostructures are being explored by researchers to detect biomolecules at sensitivities beyond the picomolar range. It is recognized, however, that nanostructuring of surfaces alone is not sufficient to enhance sensor sensitivities down to the femtomolar level. In this paper, we break this barrier/limit by introducing a sensing platform that uses a multi-length-scale electrode architecture consisting of 3D printed silver micropillars decorated with graphene nanoflakes and use it to demonstrate the detection of dopamine at a limit-of-detection of 500 attomoles. The graphene provides a high surface area at nanoscale, while micropillar array accelerates the interaction of diffusing analyte molecules with the electrode at low concentrations. The hierarchical electrode architecture introduced in this work opens the possibility of detecting biomolecules at ultralow concentrations.

## Abstract (web summary)

Here, the authors introduce a biosensing platform with multi-length-scale electrode architecture consisting of 3D printed silver micropillars decorated with graphene flakes. They demonstrate that this breaks a barrier to the biomolecule limit-of-detection, enabling detection down to femtomolar level.

## Introduction

One of the key requirements for the effective management and treatment of diseases is their detection at an early stage. This requires a timely detection of the relevant biomarkers at very low concentrations. Despite considerable progress in this area, sensing techniques of broad applicability that can detect a range of biomarkers at ultralow (e.g., femtomolar or attomolar) concentrations with high throughput (e.g., detection time in seconds or minutes) are yet to be realized^1,2^. This limitation has created economic burdens that were apparent in worldwide ravage caused by the pandemic associated with the novel coronavirus^2^. Most importantly, the human cost of this limitation needs to be addressed with utmost urgency.

Electrochemical transduction is a well-established surface-sensitive technique to detect biomolecules of various types^3^. An obvious method to increase its sensitivity is the nanostructuring of the surfaces, which is expected to increase the surface area of the reaction^4–8^. Several nanostructured surfaces have been explored for this purpose such as DNA nanostructures^9^, Bi_2_S_3_ nanowire microfibers^10^, Au-Pd/MoS_2_-MWCNT nanocomposite^11^, NiCo_2_S_4_ nanoparticles^12^, and host-surface nanotexturing^8,13^. These approaches have led to a significant advance in the detection sensitivity. However, as pointed out in the seminal works by Sheehan and Whitman^1^ and others^7,14–16^, the nanostructures are typically grown on two-dimensional (2D) surfaces, making it challenging for relevant analyte molecules to ‘collide’ with them at practically relevant time scales. This is especially true when the concentration is low, typically below about picomolar level. In one approach to overcome this issue, antibodies attached to magnetic particles are directed to sorting wells under a magnetic field to detect protein molecules at subfemtomolar concentrations^17^. Other suggested approaches include a multi-length scale electrode architecture, where the surfaces of micro and mesoscale structures enable an active interaction of the analyte molecules even at low concentrations, while nanostructures on the electrode surface give the surface enhancement that can lower the limit-of-detection (LoD) for sensing in a reasonable amount of time^14^.

The emergence of additive manufacturing, i.e., three-dimensional (3D) printing, has allowed the fabrication of micro-to-mesoscale multi-level 3D device architectures that were difficult, if not impossible, to build via conventional lithography. This was made possible via the recent work in our group where Aerosol Jet (AJ) nanoparticle printing (a method previously used to make 2D electronic devices^18^) was used to create 3D complex device architectures with solid truss members leading to aspect ratios >20:1^19^. The 3D printed 3D geometry enables an electrode architecture that has multiple hierarchical length-scales (e.g., micro to mesoscale). Another feature of 3D printing is that different material types can be seamlessly integrated with the complex architecture being printed. For example, atomically thin nanomaterials such as graphene flakes can be combined with the electrode to enable ultra-high surface area in addition to the micro-patterning achieved by printing. Lastly, since the printed microarchitectures are typically formed on a substrate^19^, they can be combined with microfluidic devices where analyte molecules can be conveniently introduced into the fluid flow of the device.

Dopamine is one of the biomolecules that can be detected at low concentrations with significant clinical advantages^20^. It is an electrochemical messenger in the brain and controls motor coordination, emotions, cognition, and reward-motivated behavior^21^. Inadequate levels of dopamine can cause vasodilation outside of brain, attention deficit hyperactivity disorder (ADHD), dementia with Lewy bodies, and Huntington’s disease, while its high level is associated with brain cancers such as neuroblastoma^21,22^. Though the physiological range of dopamine in human biofluids (blood, urine, cerebrospinal) are different, according to Human Metabolome Database, the dopamine concentration in blood is <130 pm (see Supplementary Table 1)^23^. Prior reports on the detection of dopamine at nanomolar (nm) concentration do exist^20,24,25^. For example, an electrochemically grown reduced graphene oxide (rGO)-based electrode has been utilized to detect dopamine at 0.1 µm via direct catalytic oxidation reaction^26^. All these methods involve nanomaterials on 2D surfaces to increase the detection sensitivity. Other dopamine detection methods include enzyme-linked immunosorbent assays (ELISA) (~1.26 nm)^27^, high-performance liquid chromatography (~65.3 pm)^28^, ultraviolet spectroscopy (~1 μm)^29^, fluorometry assays (~20 nm)^30^, electrochemiluminescent (100 nm)^31^, 2D-material-based electrochemical sensors (50 pm)^20^, porphyrin-based metal–organic framework nanocrystals coated poly(3,4-ethylenedioxythiophene) (40 nm)^32^, and enzyme-based electrochemical sensors (1 nm)^33,34^.

In this work, we are motivated by the exciting possibility of creating a nano-to-mesoscale biosensing platform, envisioned in the theoretical work in literature^1^, that overcomes the limits of the current electrode architectures to achieve femtomolar level of LoD. The focus is on creating a multi-length-scale electrode architecture consisting of AJ printed micro- and mesoscale hollow silver pillars that ‘protrude’ into the microfluidic channel containing the analyte, dopamine, and, coating the electrodes with atomically thin sheets of rGO. The multi-scale architecture is expected to accelerate the electrochemical redox reaction by allowing a more effective interaction of the analyte molecules (i.e., dopamine) with the atomically thin rGO to achieve a sub-picomolar LoD. By nanostructuring the surface and controlling multi-length-scale electrode structure, the LoD for dopamine was tuned from micromolar to femtomolar concentration, a significant result for clinically relevant diseases. This construction is also designed to enable an enzyme-free detection of dopamine with a wide linear measurement range (fm to nm range). Lastly, the architecture is expected to facilitate the introduction of perfluorinated polymer layer, or Nafion, into the electrode, enhancing the selectivity of the sensor by eliminating negatively charged interfering molecules.

## Results and discussion

### AJ nanoparticle printing of the electrodes for the biosensing platform

**Fig. 1:**
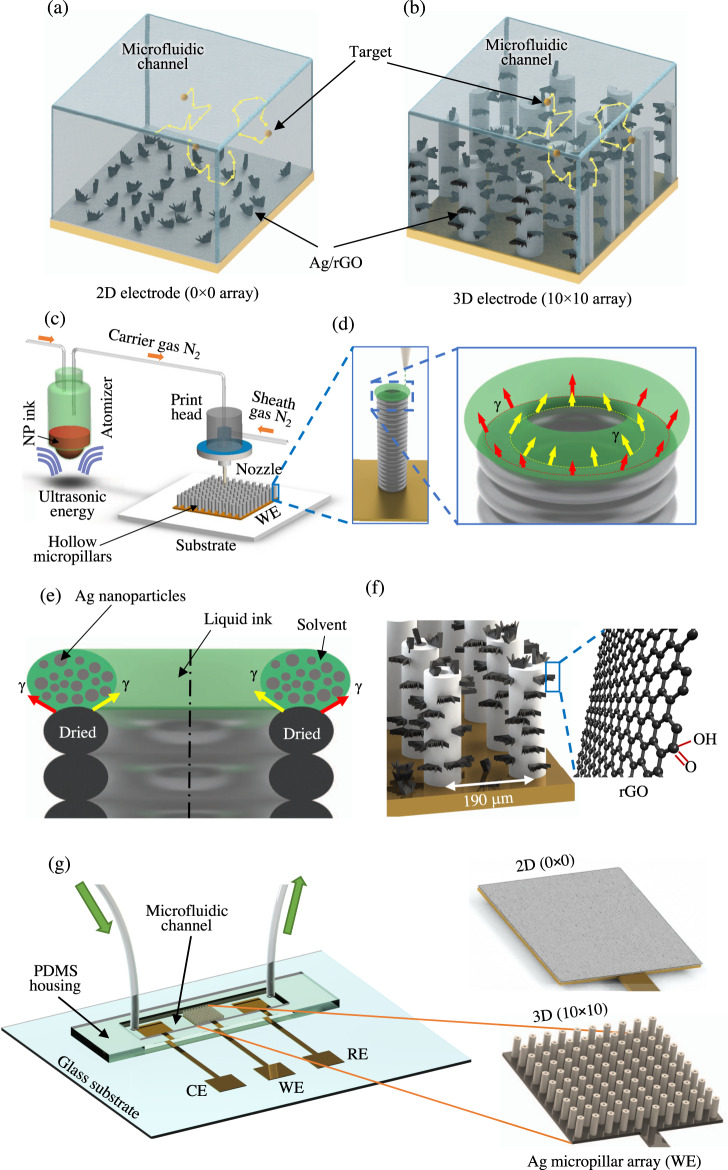
Aerosol Jet nanoparticle 3D printing process for micropillar array electrodes used for dopamine sensing. **a**, **b** Schematic representations of Brownian-motion of target molecules for a 2D electrode (0 × 0 array) and a 3D electrode (10 × 10 array) within the microfluidic chamber. **c** AJ 3D printer with an ultrasonic atomizer and a print head. The metal nanoparticle ink is converted into aerosol of ink droplets in the atomizer. It is then carried to the nozzle via a tube using a carrier gas (N_2_). A sheath gas, also N_2_, focuses the aerosol on the substrate at a length scale of 10 μm. **d** The mechanism of hollow-micropillar formation with concentric toroid-shaped rings of metal nanoparticle (NP) ink printed layer-by-layer. The red and yellow arrows show the surface tension of the liquid ring which helps in the buildup of the structure. Once one layer is printed, it loses solvents due to the heat from the platen and provides a solid base for the next layer of toroid-shaped ring of the ink to be printed. **e** Cross-sectional view of the printing process shown in (**d**) where surface tension, γ, provides the fluid dynamic stability required to keep the ring in a stable state. The top liquid ring contains silver nanoparticles with solvents and binders before losing the solvents due to the heat from the platen. The solidified structure is then sintered in an oven as discussed in the “Methods” section. **f** A schematic showing the silver micropillars with rGO flakes attached to their surfaces. **g** Schematic of the dopamine sensing device including the PDMS housing, a microfluidic channel, a 3D printed micropillar array (from (**c**)), tubes for injection and removal of dopamine solution (RE-reference electrode, CE-counter electrode, WE-working electrode). Schematic of a 2D silver sensor fabricated by AJ printing method and used to assess a comparative performance is also shown.

We first describe the AJ nanoparticle printing method used to fabricate the multi-length-scale biosensing platform developed in this work. Figure 1 shows the schematics of the mechanism of target molecule capture in the biosensor and the sensor device and its fabrication via the AJ nanoparticle printing method. Figures 1a, b show the schematics of 2D and 3D electrode configurations, respectively, along with the mechanism of the capture of the target molecules by the 3D electrode architecture. The 3D micropillar electrode array is expected to facilitate the interaction of the target molecules with the electrode surface, leading to low LoD for detecting biomarkers within reasonable detection time. Figure 1c shows the working principle of the AJ printer, which involves breaking of a nanoparticle ink solution stored in a vial into micron-sized droplets using ultrasonic energy and carrying them to a nozzle by an inert gas, followed by dispensing on a heated substrate (see the “Methods” section for details). Figure 1d shows dispensing a toroid-shaped layer of the condensed silver nanoparticle ink on a platen that was heated to a temperature of 100 °C. As soon as one layer was printed, the solvent evaporated due to the heat from the platen, forming a ring of solidified ‘dry’ material containing nanoparticles and binders. When the next ring was printed, the surface tension of the toroid-shaped ring (indicated by red and yellow arrows) allowed the pillar to be built as shown in Fig. 1e. Each layer of the toroid-shaped ring was about 5–10 μm thick and formed within a fraction of a second. The heating of the dried micropillar array removed the binders and sintered the nanoparticles to create the 3D electrode array. Note that the printing method shown in Fig. 1d, e uses aerosol droplet dynamics to form the 3D electrodes and does not depend on the chemistry of metal nanoparticles in the ink. As a result, the construction method for the 3D micropillars is applicable to other functional materials, as long as the corresponding nanoparticle inks are being used. Figure 1f shows the rGO flakes that adhere to the silver micropillar electrodes. The exfoliated rGO sheets partially cover the micropillar surfaces. However, multiple atomically thin sheets may interact with adjacent ones due to strong π-π interactions among the graphene sheets.

Figure 1g shows a schematic of the sensing platform which consists of three electrodes (counter electrode or CE, reference electrode or RE, and working electrode or WE) on a glass substrate, and a PDMS housing that forms the electrochemical device. The 3D micropillar electrode described in Fig. 1d, e was directly printed on the WE. Note that based on the 3D construct of the electrode, the increase of the physical area for the micropillar structures over a planar electrode is about 260%. Also, as shown in Fig. 1g, a 2D silver block electrode with the same base area (2 mm × 2 mm) as the 3D micropillar array and with 50 µm height was fabricated using AJ printing on WE for comparison with the 3D electrodes. Reduced graphene oxide flakes/sheets dispersed in water (1 mg/mL) and containing 5% Nafion were used to coat the 2D silver and 3D silver micropillar electrodes to detect dopamine via electro-oxidation mechanism. Note that the surface of silver microelectrode is known to bind with rGO nanoflakes via π-π or electrostatic interactions^35^. We decided to explore sintered silver microelectrodes decorated with rGO nanoflakes for the sensing platform because of the excellent electro-catalytic properties demonstrated by these materials in previously reported literature^36^.

**Fig. 2:**
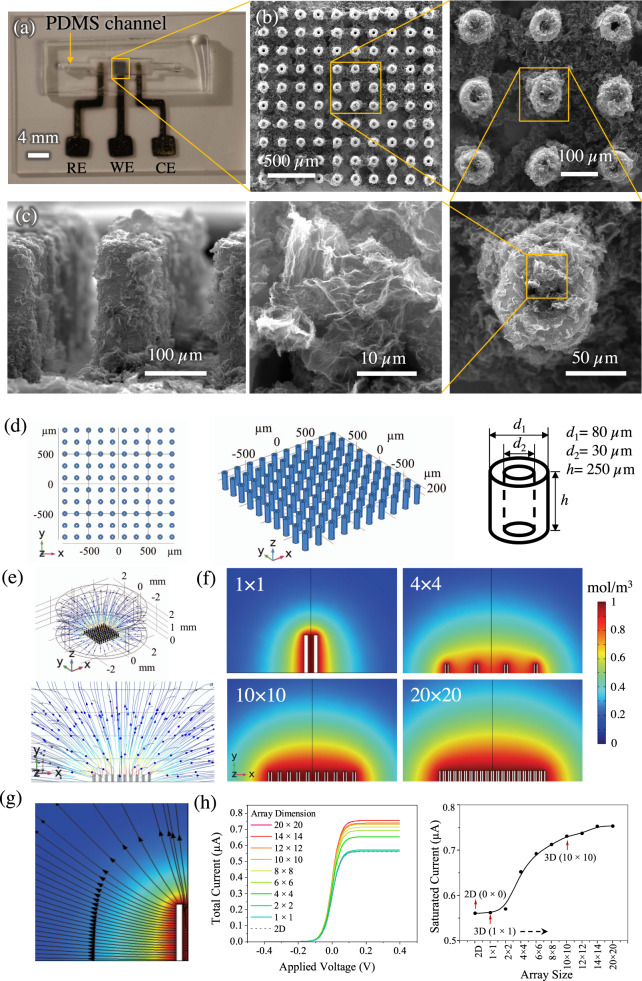
The nano-to-meso multi-length-scale AJ printed biosensing platform. **a** Optical image of the microfluidic sensing platform for dopamine detection consisting of the electrodes and the PDMS housing. **b** SEM images of the AJ printed 3D Ag/rGO micropillar array electrode at different magnifications. The top-down view of the micropillars with inner diameter of 28 μm and outer diameter of 80 μm is shown in the micrographs at an increasing magnification. The rGO flakes attached to the silver micropillar surface are also observed. **c** The side-view of the Ag/rGO micropillar array showing the rGO coating and dimension of the pillar. Two repeated measurements of Fig. 2b, c were performed on two separate sensors. **d**–**h** COMSOL simulation of the electrochemical cell. **d** Model geometry with top-view, 3D perspective view, and dimensions for 10 × 10 configuration. **e** Characteristic diffusion profiles for oxidation of redox species for 10 × 10 micropillar array configuration for the electrode. **f** Concentration profiles of diffusing species at the central plane for different arrays of the electrodes—1 × 1, 4 × 4, 10 × 10 and 20 × 20. **g** Mass flux streamlines for half of a single hollow micropillar (cross-section) which shows the contribution of planar and radial diffusion of electro-species. **h** One-dimensional (1D) plots of total current obtained from different array configurations varying from 1 × 1 to 20 × 20 as a function of applied voltage (V). The saturation current is obtained for each array configuration. Data for 2D configuration (0 × 0 array) is also included. The data show that for an array with configuration denser than 10 × 10 in the microfluidic chamber, the saturation current does not increase further. The electrode configuration of 10 × 10 array was chosen for this study.

Figure 2a–c shows scanning electron microscopy (SEM) images of representative 3D 10 × 10 micropillar array electrodes coated with rGO sheets and integrated in the microfluidic channel, forming the sensing platform. Figure 2a shows the PDMS mold placed on the three electrodes (RE, WE, and CE) creating the microfluidic chamber for the electrochemical detection of dopamine. Supplementary Fig. 1 shows the fabrication process of the PDMS microfluidic device using the replica molding method. Each micropillar had inner and outer diameters of 28 μm and 80 ± 1.39 μm, respectively, and a height of 239 ± 5.5 μm, while the pillar-to-pillar distance was 190 ± 0.1 μm (see Supplementary Fig. 2a–c). The outer diameter of the micropillar varied between 74 and 81 μm. This variation likely came from the fact that during printing, the bottom portion of the pillar experienced the highest amount of thermal energy from the platen which led to a higher volumetric shrinkage. This effect, however, faded rapidly as the pillar was being built and the outer diameter quickly approached to a steady value, which was about 80 μm in the present case. From Fig. 2b, c, the rGO nanoflakes are seen to be adhered on the silver surface. However, in some cases, more flakes were seen on the top surface of the pillar compared to the sides. Supplementary Fig. 3 shows the SEM images of a 4 × 4 array evaluated in this work (before the coating of the rGO flakes). As stated before, we also printed and sintered 2D silver electrodes for comparison with 3D Ag/rGO micropillar electrode architectures (10 × 10 and 4 × 4 micropillar arrangements). SEM images of the 2D silver electrode are shown in Supplementary Fig. 4. As shown in Figs. 1 and 2, the micropillar array increased the active surface area of the sensor by >260% when compared to a planar 2D electrode. The increase in the surface area alone, however, is not sufficient for large-scale improvement in LoD as noted in prior literature^1^. As shown in Fig. 1, the micropillar geometry is expected to facilitate an interaction of the dopamine molecules with the nanostructured electrode surface. The choice of the 10 × 10 micropillar array and the hollow geometry for the electrode (Fig. 2b, c) was made using a COMSOL simulation of the sensing process which is discussed next.

The benefit of the 3D electrode geometry for the sensing platform as demonstrated in this research can be assessed by first modeling the electrochemical processes with hollow micropillar array configuration. To achieve this, a 3D simulation was conducted using the electroanalysis module of the COMSOL Multiphysics^®^ software. Figure 2d–h shows the model set-up and the simulation results. Figure 2d shows the model of the 10 × 10 micropillar array geometry with top and 3D perspective views, along with individual pillar dimensions. Note that several other configurations— from 0 × 0 to 20 × 20 pillars—were also analyzed. The diffusion profiles for the 10 × 10 configuration is shown in Fig. 2e. Figure 2f shows the concentration of the diffusing species for 1 × 1, 4 × 4, 10 × 10, and 20 × 20 pillar arrays. The diffusion profile for 2D (0 × 0) electrode is shown in Supplementary Fig. 5. It is known that linear diffusion of the species occurs when the electrode has a 2D planar geometry resulting in a relatively low redox current^37^. Unlike the planar electrodes, the 3D cylindrical electrodes used in this work show a uniform radial diffusion in all directions combined with linear diffusion (Fig. 2g). The arrangement also offers an enhanced possibility of analyte molecules interacting with the electrode surface when compared to 2D surfaces. Interestingly, the hollow geometry of the pillars enabled an enhanced diffusion inside the cavity of the pillar electrodes as well (Fig. 2g)—providing an added advantage not offered by solid pillars. As the array size was increased, a significant enhancement in total current is observed (Fig. 2h). This increase, however, reached a plateau when the array size was increased beyond 10 × 10. We note that in Fig. 2h, the individual micropillar size had to be reduced to fit the array within the geometry of the microfluidic device for the arrays larger than 10 × 10. Based on the results presented in Fig. 2b, c and Supplementary Fig. 5, we chose the optimum micropillar array configuration of 10 × 10 (3D) for the development of the sensing platform in this work. In addition, for comparison, we also experimented with 4 × 4 (3D) array and 0 × 0 (2D) array electrodes (Supplementary Figs. 3 and 4).

### Electrochemical characterization of the multi-scale sensing platform

**Fig. 3:**
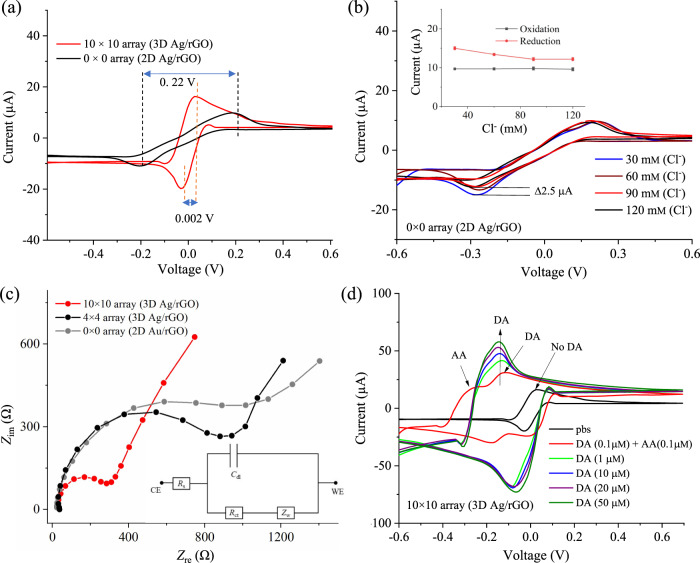
Cyclic voltammetry results for different electrode architectures. **a** CV of 2D Ag/rGO (0 × 0 array) and 3D Ag/rGO (10 × 10 array) electrodes at a fixed scan rate of 0.5 V/s. The CV experiment was carried out in the presence of pbs (50 mm, pH 7.4) added with equimolar concentration (1.0 mm) of ferro/ferricyanide [Fe(CN)_6_]^3−/4−^mediator. **b** Effect of chloride (Cl^−^) concentration using CV measurements for 2D Ag/rGO (0 × 0 array) electrode where the Cl^−^ is varied from 30 to 120 mm. Inset of (**b**) depicts the variation of oxidation/reduction peak currents with Cl^−^ concentrations. The concentration of ferro/ferricyanide was set to 1 mm at a fixed scan rate of 0.5 V/s for this study. Oxidation current is found to be unchanged, but reduction potential showed a minute variation of current, ~2.0 µA, at Cl^−^ concentration of 30–90 mm after which it becomes saturated and stable. The graph in inset (**b**) corresponds to the median value of three repeated measurements (*n* = 3). Error bars: median ± SD. **c** The electrochemical impedance spectroscopy (EIS) responses of 2D Au/rGO (0 × 0 array), 3D Ag/rGO (4 × 4 array), and 3D Ag/rGO (10 × 10 array) for a voltage amplitude of 1 mV as a function of the frequency (1–10,000 Hz). Inset of (**c**) shows the equivalent electrical circuit diagram to evaluate the charge transfer resistance (*R*_ct_). The EIS/CV experiments were conducted in presence of pbs (50 mm, pH 7.4) containing an equimolar concentration of 1 mm [Fe(CN)_6_]^3−/4−^. **d** 3D sensor (10 × 10 array) electrode was used to detect dopamine concentration of 0.1–50 µm and an investigation of cross-reactivity effect was performed in presence of ascorbic acid (AA) (0.1 µm) at a fixed scan rate (0.5 V/s) and Cl^-^ concentration (120 mm). The results indicate that the current increases with an increase in dopamine concentration at a fixed oxidation potential of −0.14 V.

Cyclic voltammetry (CV) studies were carried out to investigate the electrochemical behavior of 2D silver and 3D silver micropillar geometries coated with rGO as shown in Fig. 3. The CV graphs for a planar 2D Ag/rGO electrode (0 × 0 array) and a 3D Ag/rGO electrode (10 × 10 array) are shown in Fig. 3a. Note that the base area of the planar electrodes was approximately the same as the projected area of the 3D micropillar electrodes. The CV tests were carried out in the presence of phosphate-buffered saline (pbs) solution with 1 mm solution of ferro/ferricyanide ([Fe(CN)_6_]^3−/4−^) redox mediator (Fig. 3a). The CV curves demonstrate excellent oxidation and reduction peaks of the redox species for both electrode configurations indicating that the electrodes exhibited surface-controlled process. The sensor with 3D Ag/rGO micropillar architecture showed a superior oxidation current (16 µA) compared to the sensor with 2D Ag/rGO (9 µA) electrode (Fig. 3a). Compared to 2D Ag/rGO electrode (0.22 V), the 3D Ag/rGO electrode showed a lower peak-to-peak separation voltage (0.002 V) indicating high electron transfer kinetics (Supplementary Table 2). Clearly, the higher oxidation current for the AJ printed 3D Ag/rGO electrode compared to 2D Ag/rGO electrode is due to the increased surface area and the enhanced diffusion of electroactive species from the electrolyte enabled by the cylindrical geometry of the micropillars (also indicated by the COMSOL simulations shown in Fig. 2). We note that Garg et al.^38^ have indicated the possibility of silver nanoparticles reacting with inorganic ligands such as chloride (Cl^−^) via electrochemical oxidation during measurements, potentially reducing the current. In the current work, however, it is expected that the protonated Nafion and multiple layers of rGO covering the silver electrodes prevent the Cl^−^ ions from reacting with silver. To verify this, we measured the effect of Cl^−^ concentration on a base electrode made of silver nanoparticles as shown in Fig. 3b. Results of 2D Ag/rGO electrode show that the oxidation current remains unchanged with Cl^−^ concentration while the reduction has a minute change (2 µA). In fact, for the physiological range of Cl^−^ concentration in human serum (96–106 mm), the change in current is insignificant. We thus sense dopamine by taking advantage of the fixed oxidation potential of our 3D printed device.

To obtain a deeper insight into the advantages offered by the AJ printed 3D Ag/rGO electrode geometry, we also carried out electrochemical impedance spectroscopy (EIS) at a frequency range of 1–10,000 Hz to investigate the charge transfer resistances (*R*_ct_) at the electrode-electrolyte interfaces. Figure 3c shows the Nyquist plots of 3D Ag/rGO (10 × 10), 3D Ag/rGO (4 × 4), and 2D Au/rGO (0 × 0) electrodes (the last case obtained for comparison purposes without the printing of silver on the evaporated Au electrode). The values of *R*_ct_ estimated from these plots (Fig. 3c) by comparing it with an ideal equivalent circuit (shown in the inset of Fig. 3c) are 0.267, 0.887, and 1.004 kΩ, respectively. At low frequencies, the electrodes show diffusion-limited behavior, while at higher frequencies, they show a charge-transfer-limited behavior as indicated by the straight line and the diameter of semicircle (*R*_ct_), respectively. The *R*_ct_ and the double-layer capacitance (*C*_dl_) are obtained from the real (*Z*_re_) and imaginary (*Z*_im_) parts of complex impedance at different frequencies for a parallel RC circuit given by^39^,

1 $$Z(\omega)=\; 	{R}_{s}+\frac{{R}_{{{{{{\rm{ct}}}}}}}}{1+j\omega {R}_{{{{{{\rm{ct}}}}}}}{C}_{dl}}={R}_{s}+\frac{{R}_{{{{{{\rm{ct}}}}}}}}{1+{\omega }^{2}{R}_{{{{{{\rm{ct}}}}}}}^{2}{C}_{{{{{{\rm{dl}}}}}}}^{2}}\\ 	 +\frac{j\omega {R}_{{{{{{\rm{ct}}}}}}}^{2}{C}_{{{{{{\rm{dl}}}}}}}}{1+{\omega }^{2}{R}_{{{{{{\rm{ct}}}}}}}^{2}{C}_{{{{{{\rm{dl}}}}}}}^{2}}={Z}_{{{{{{\rm{re}}}}}}}+j{Z}_{{{{{{\rm{im}}}}}}}$$

Here *R*_*s*_ is the solution resistance. The heterogeneous electron transfer (HET) rate constant (*k*_0_) for all the electrode geometries is defined^39^ as

2 $${k}_{0}=\frac{RT}{{n}^{2}{F}^{2}AC_{0}{R}_{ct}},$$

where *T* is the temperature (298 K), *R* is the gas constant (8.314 J/K mol), *n* is the number of electrons that are transferred in the redox couple (1), *F* is Faraday’s constant (96485.3 C/mol), *A* is the area of the electrode, and *C*_*0*_ is the bulk concentration of the redox species (1 mm). Interestingly, the dramatic reduction of *R*_ct_ for 3D Ag/rGO micropillar electrodes could be due to both the linear and spherical diffusion of electrons surrounding the 3D geometry of micropillars from bulk electrolyte, leading to a higher electron transfer rate (*k*_0_) compared to 2D Ag/rGO electrode (see Supplementary Fig. 6). The sluggish and linear diffusion of electro-species resulted in higher *R*_ct_ values for 2D Ag/rGO and 2D Au/rGO electrodes compared to 3D Ag/rGO micropillar electrode. The diffusion co-efficient of the electrodes are estimated using Randles-Sevcik equation by a method demonstrated in literature^40^. The effective diffusion co-efficient (*D*_*0*_) for both 3D Ag/rGO and 2D Ag/rGO electrodes were evaluated. The value of *D* for the AJ printed 3D silver micropillar electrodes was higher than that for planar geometries (Supplementary Table 2). The 3D electrode structure is speculated to establish an electronic conduction pathway, which reduces the tunneling distance for the diffusing [Fe(CN)_6_]^3−/4−^ ions (or electrons) to the current collector. In addition, in the presence of low concentration of dopamine molecules, the 3D geometry will enhance the interaction of the analyte molecules with the electrode. We thus confirm that the sensing platform developed in this work enhances reactivity of the redox species as predicted in the COMSOL simulations in Fig. 2d–h.

**Fig. 4:**
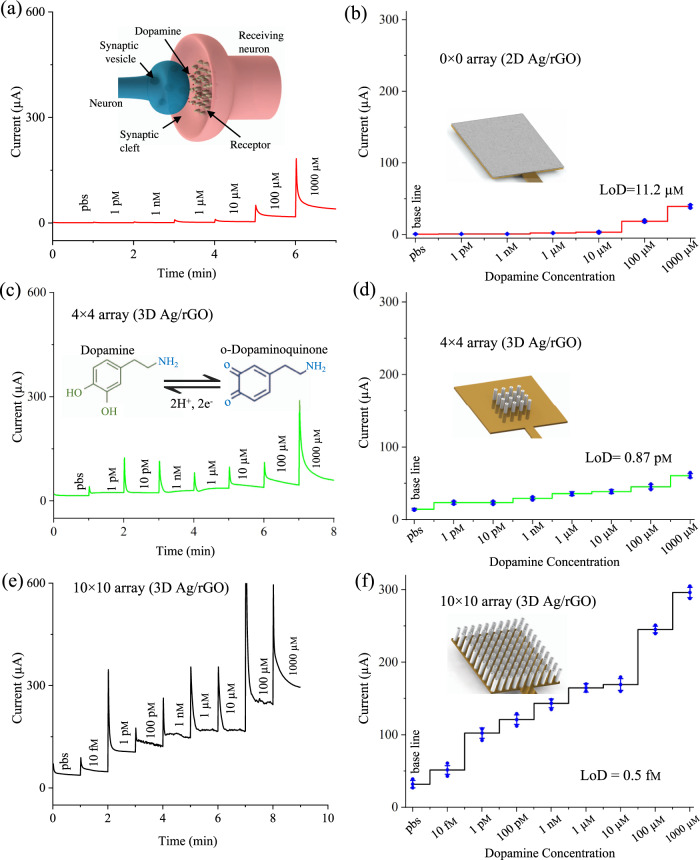
Sensing of dopamine molecules. **a** Chronoamperometric sensing curves for 2D Ag/rGO (0 × 0 array) sensor. The dopamine (dopamine hydrochloride) concentration is diluted from 1 pm to 1000 μm in pbs solution (pH 7.4) containing 1 mm concentration of ferro/ferricyanide. The measurements were conducted without any enzyme at a fixed sensing potential of −0.1 V for 1 min. Inset of (**a**) shows a schematic of dopamine molecules released from pre-synaptic neuron to target post-synaptic neuron. **b** Saturation currents of 2D Ag/rGO sensor plotted against varying dopamine concentrations. **c**, **e** Chronoamperometric sensing of multi-length-scale 3D Ag/rGO (4 × 4 array) and 3D Ag/rGO (10 × 10 array) sensors, respectively, at the same condition as that performed for 2D Ag/rGO sensor. A low concentration (10 fm) of dopamine was tested in addition to 1 pm to 1000 μm for 3D Ag/rGO (10 × 10 array) sensor. Inset of (**c**) shows the biochemical oxidation/reduction reaction of dopamine. **d**, **f** Current responses with varying dopamine concentration for multi-scale 3D Ag/rGO (4 × 4 array) and 3D Ag/rGO (10 × 10 array) configurations, respectively. The detection time for all the results in this figure are within 60 seconds. Error bars in Fig. 4b, d, f are the standard deviation of three repeated measurements (*n* = 3 biologically independent experiments). Error bars, mean ± SD.

Once we established the enhanced reactivity of the redox species in the 3D geometry, the 3D Ag/rGO micropillar sensor was tested using CV technique in presence of ascorbic acid (0.1 µm) and dopamine (0.1 µm) (Fig. 3d) at the same scan rate of 0.5 V/s. This experiment also led us to find the sensing potential of dopamine molecules for the chronoamperometric experiments to be carried out later-on. From Fig. 3d, the sensor with 3D Ag/rGO micropillar electrodes shows a distinct redox curve with oxidation and reduction peaks due to the ferro/ferricyanide redox mediator. Upon introduction of ascorbic acid and dopamine molecules, the redox peak current is found to increase and is shifted to a lower potential. Note that the two distinct redox peaks appeared due to the presence of dopamine and ascorbic acid molecules. Further, when we increased the concentration of dopamine from 0.1 to 50 µm, the side peak disappeared as we did not add any ascorbic acid molecules. Thus, it is concluded that the oxidation potentials such as −0.14 and −0.22 V are responsible for oxidizing dopamine and ascorbic acid, respectively. On addition of dopamine molecules, the CV showed an excellent dominant redox peak at −0.14 V due to its direct catalytic conversion of dopamine to o-dopmanoquinone and two electrons (2e^−^) and two protons (2H^+^)^41^. The biochemical reaction of dopamine molecules is shown in the inset of Fig. 4c. Thus, this result indicates that the sintered silver nanoparticles in the microelectrodes coated with rGO acted as detection probe for the catalytic conversion of dopamine without any supplementary dopamine-specific enzyme molecules, which is important to avoid biofouling^42,43^. As the oxidation potential (−0.14 V) is selective to dopamine only (w. r. t. ascorbic acid), we chose this potential (−0.14 V) for the sensing of dopamine in the next experiments.

### Dopamine detection using the AJ printed multi-length-scale sensing platform

Once the oxidation potential for the enzyme-free dopamine detection was obtained, chronoamperometric studies were carried out for the 2D Ag/rGO (0 × 0 array), 3D Ag/rGO (4 × 4 array), and 3D Ag/rGO (10 × 10 array) sensors. Before dopamine sensing, the baseline of three sensors was obtained using chronoamperometric method at a fixed potential of −0.14 V (Supplementary Fig. 7). The base line of 3D Ag/rGO (10 × 10 array) sensor is found to be higher (37 µA) than the other sensors such as 3D Ag/rGO (4 × 4; 15 µA) and 2D Ag/rGO (0 × 0; 0.8 µA). This is due to the higher number of pillars with 3D structures which can provide more surface area for redox reactions.

The dopamine sensing was based on the principle of chronoamperometric method wherein the current of the micropillar array electrode was measured at a fixed sensing potential with respect to the RE in presence of a variety of electrolyte solutions. The resulting current at the WE due to the faradaic process for a fixed potential step was recorded as a function of time. During sensing of dopamine, the fixed potential was stepped to a value where the dopamine showed a redox behavior. According to the Cottrell equation, the current decays with the square root of time (t^1/2^) under a diffusion control process and is given by,

3 $$i(t)=\frac{nFA{D}_{0}^{1/2}{C}_{0}}{{\pi }^{1/2}{t}^{1/2}}$$

where *n* is the number of electrons, *A* is the electrode area, *F* is the Faraday constant, *D*_*0*_ is the diffusion co-efficient of the redox species, *t* is the time, and *C*_*0*_ is the bulk concentration of the redox species. Although the current paper focuses on in vitro sensing of dopamine, we depict the dopamine transfer through the synaptic cleft between pre-synaptic neuron to post-synaptic neuron via ligand-gated ion channels as presented in Fig. 4a, which is the neurotransmitter of interest.

Figure 4a–f shows the representative graphs of 2D Ag/rGO (0 × 0 array), 3D Ag/rGO (4 × 4 array), and 3D Ag/rGO (10 × 10 array) sensors to detect dopamine at concentrations from 10 fm to 1000 μm. During detection, each concentration of dopamine was introduced into the microfluidic sensor via a pre-loaded syringe and measurements were conducted in static conditions. Once the fluid was loaded onto the chip, the measurement was taken immediately without any incubation time (within 60 s). The sensor was washed using pbs solution before exposing a new solution of dopamine for the next chronoamperometric measurements. As the dopamine concentration increased, it was observed that the output current varied for both sensors. This change is attributed to catalytic oxidation of dopamine resulting in the formation of dopamine-o-quinone, and two electrons (inset of Fig. 4c). During the catalytic conversion of dopamine, the positively charged dopamine^44^ molecules in pbs solution at a pH 7.4 bind with negatively charged Ag/rGO micropillar surface^45^ via electrostatic interactions and facilitate direct electron transfer toward current collector.

The current densities for the sensor data in Fig. 4 are plotted in Supplementary Fig. 8. It is noted that the ratio of the areas of 3D Ag/rGO (10 × 10), 3D Ag/rGO (4 × 4), and 2D Ag/rGO (0 × 0) sensor configurations is 2.6:1.6:1. The current densities at 1 pm concentration of dopamine for all these configurations such as 3D (10 × 10), 3D (4 × 4), and 2D (0 × 0) are measured as 41.7:15.5:1 (Supplementary Fig. 8). Though the physical surface area is increased by 260% for the 3D (10 × 10) sensor with respect to the 2D sensor, the resulting current of 3D (10 × 10) sensor is increased by 4170% at 1 pm concentration of dopamine. As discussed, this significant enhancement of current with the 3D configured sensor is due not only to the enhancement in the surface area of the electrode, but also to the multi-length scale architecture that leads to spherical diffusion of analyte molecules as shown in the COMSOL simulations. Supplementary Fig. 9 shows the calibration results (Supplementary Fig. 9a–c) and comparison plots (Supplementary Fig. 9d, e) for the current obtained for the three sensors and plotted as a function of the dopamine concentration.

The 3D Ag/rGO sensor with 10 × 10 array configuration showed two linear responses for dopamine; first response is from 10 fm to 10 μm, and the other response is from 10 μm to 1000 μm concentration of dopamine with slope values given in Supplementary Fig. 9c (with *r*^2^ of 0.99 and 0.95, respectively). Similarly, the 3D sensor with 4 × 4 array also showed two linear response regions for dopamine concentration (Supplementary Fig. 9b) from 1 pm to 10 μm (*r*^2 ^= 0.994), and 10 to 1000 μm (*r*^2 ^= 0.97) concentration of dopamine. However, the 2D sensor with 0 × 0 array showed only one linear response region (Supplementary Fig. 9a) from 10 to 1000 μm (*r*^2 ^= 0.982) concentration of dopamine. Their analytical sensitivities are found to be 1 μm (0 × 0), 10 pm (4 × 4), and 10 fm (10 × 10). The slope values are found to be higher in the case of 3D sensor with 10 × 10 geometry. LoD is determined for all three sensors by a method reported in literature^46,47^, and calculation is shown in Supplementary Section 1. Using 3D Ag/rGO micropillar array in this device of 10 × 10 configuration, we were able to achieve a low LoD of 0.5 fm and a wide detection range of 10 fm–10 μm for dopamine compared to other sensor configurations such as 4 × 4 array (0.87 pm) and 0 × 0 array (11.2 µm). In addition, the analytical sensitivities are found to be 1 µm, 1 pm and 10 fm for the three sensor configurations of 2D Ag/rGO (0 × 0 array), 3D Ag/rGO (4 × 4 array), and 3D Ag/rGO (10 × 10 array), respectively. These results clearly demonstrate the superior performance obtained by using 3D architectures for the electrodes in the electrochemical cell.

Table 1 compares the dopamine detection results shown in Fig. 4 with those reported in literature in a variety of biological media. The LoD for dopamine sensing reported in this work is lower than any other study in literature. In fact, compared to using different materials such as graphene^48^, nano-gold^49^, planar carbon paste^50^, 3D carbon^51^, 2D plasmonic hole array^52^, nickel oxide nanoparticles^34^, and planar gold (Au)-CoP^53^, the use of 3D Ag/rGO micropillar geometry with its nano-to-meso scale architecture improved the LoD for dopamine detection by about nine orders of magnitude. As the measured physical range of dopamine in human blood and plasma is as low as 0.01–0.48 nm, and 0.13 nm, respectively, the method described in this paper demonstrates not only a clear improvement over the current state-of-the-art, but also a highly useful method for neurophysiological applications.

**Table 1:** Comparison of the detection range and LoD of AJ printed 3D Ag/rGO sensor used in this work with sensors used in literature for dopamine sensing.

Materials/detection method	Detection range	LoD	Enzyme	Sensing media
2D Graphene/DPV^48^	0.5–120 μm	10 nm	No	Urine
Planar carbon paste/CV^50^	0.5–800 μm	50 nm	No	Simulated buffer
2D Nano-Au/LSV^49^	4–1012 μm	128 nm	No	pbs
3D Carbon/FSCV^51^	0.5–100 μm	10 nm	No	Mice brain (In vivo)
2D Nanoholes/Plasmonic^52^	100 fm–0.1 μm	100 fm	No	Simulated body fluid
2D Nickel oxide/CV^34^	2–100 μm	1.038 μm	Yes	Fetal bovine serum
Planar Au-CoP/DPV^53^	2–30 μm	0.43 μm	Yes	Buffer solution
PRDOT-porphyrin/DPV^32^	2–270 μm	40 nm	No	Rat brain cells (pheochromocytoma)
3D Ag/rGO (10 × 10) Array /CA	10 fm–1000 μm	0.5 fm	No	Serum (current work)

*DPV* differential pulse voltammetry, *FSCV* fast scan cyclic voltammetry, *LSV* linear square voltammetry, *CA* chronoamperometry, *CoP* cobalt (II)-porphyrin.

### In vitro tests with biological fluids

**Fig. 5:**
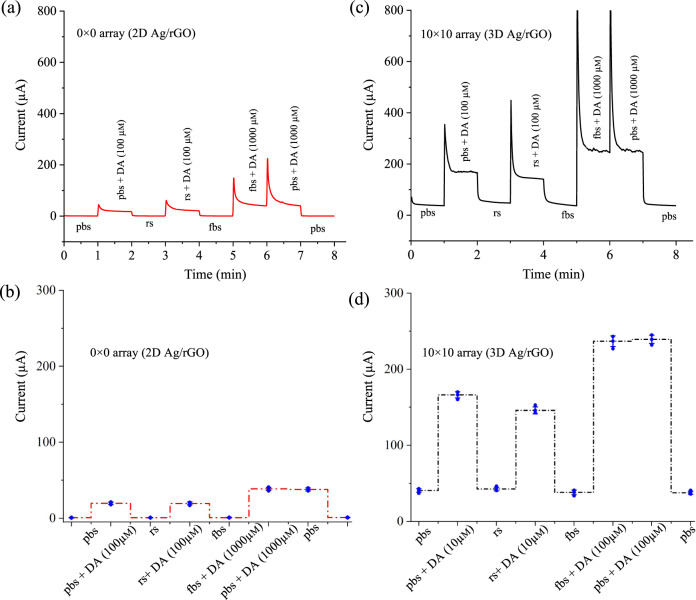
In vitro dopamine sensing. Use of chronoamperometric method to detect dopamine for (**a**) 2D Ag/rGO (0 × 0 array) and (**c**) 3D Ag/rGO (10 × 10 array) sensors in the presence of pbs, fetal bovine serum (fbs), and rabbit serum (rs) with and without the addition of different concentration of dopamine (10, 100, and 1000 μm). Both rs and fbs are diluted 15 times and mixed with dopamine concentration. **b**, **d** The chronoamperometric responses of serums with and without dopamine for both the sensors. Error bars in (**b**) and (**d**) are the standard deviation of measurements over at least *n* = 3 biologically independent experiments. Error bars, mean ± SD.

Next, we demonstrate that our multi-length-scale sensing platform can detect dopamine in vitro using biological fluids such as serum samples where we follow the protocols previously reported literature^34^. Figure 5 shows the chronoamperometric measurements of fetal bovine serum (fbs) and rabbit serum (rs) using standard addition method for both 2D Ag/rGO (0 × 0 array) and 3D Ag/rGO (10 × 10 array) sensors. For these measurements, the fbs and rs samples were diluted 15 times with pbs (pH 7.4) and were added with dopamine at 10, 100, and 1000 μm concentrations. Without dopamine, the 3D and 2D sensor signals in fbs and rs solutions are found to be very close in comparison to only pbs solution as evident by their low relative standard deviations (RSDs) of ±5.01% (3D) and ±6.12% (2D), respectively, indicating that the sensor does not show any response to a high content of proteins such as albumin or other molecules present in the serum fluids (Fig. 5b, d).

For 2D (0 × 0) sensor, on the addition of 100 μm dopamine in rs and 1000 μm dopamine in fbs, the signals are found to deviate by ±7.82% and ±1.25%, respectively, when compared to the standard 100 μm and 1000 μm dopamine in pbs solution (Fig. 5a, b). Further, for 3D multi-scale sensor (10 × 10), on the addition of 10 μm dopamine in rs and 100 μm dopamine in fbs, the signals are found to deviate by ±7.05% and ±0.41%, respectively, when compared to the standard 100 μm and 1000 μm dopamine in pbs solution (Fig. 5c, d). As the interfering protein contents are much less than in other body fluids when compared to fbs, we would expect the sensor demonstrated in this work to provide a high-fidelity performance in human biofluids such as whole blood and plasma, which we evaluate next.

**Fig. 6:**
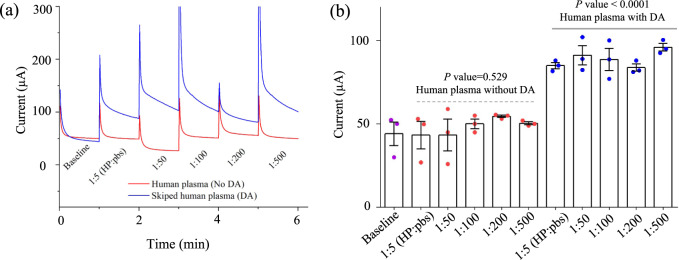
3D Ag/rGO (10 × 10 array) Sensor for the detection of dopamine in human plasma. **a** Response of the 3D printed sensing platform developed in this work for the detection of dopamine in human plasma (HP). Human plasma was diluted in pbs solution in ratios of 1:5 to 1:500 (HP:pbs). The spiked human plasma was made by adding a fixed dopamine concentration (1 pm) into all the diluted plasma. The baseline measurement was carried out without adding any plasma or dopamine. **b** Plot showing the current response (at 60s) for the data in (**a**). Error bars in (**b**) are the standard error of the mean (s.e.m.) of measurements over at least *n* = 3 biologically independent experiments. Error bars, mean ± s.e.m. Statistical significance is determined by one-way ANOVA test (non-parametric) where *p* value is 0.529 (*p* = 0.529) for human plasma without dopamine and <0.0001 (*p* < 0.0001) for human plasma with dopamine, indicating dopamine detection in HP by the 3D sensor. *p* values for each comparison are labeled on top of the graphs.

To establish the clinical relevance of the biosensing platform proposed in this work, we also conducted dopamine sensing in human plasma (HP) and artificial serum (AS) using the 3D (10 × 10) sensor. We diluted these body fluids in ratios of 1:5 to 1:500 (HP/AS:pbs) and spiked them with dopamine at 1 pm concentration. The results are summarized in Fig. 6 for HP and Supplementary Fig. 10 for AS. The chronoamperometric response of the sensor for HP with and without dopamine are shown in Fig. 6a. The sensor signal current with diluted HP without dopamine shows a deviation of ± 4.9 µA (~ ±10%; *n* = 18) from the mean signal (47.6 µA). Once dopamine is added into the diluted HP, the sensing current is increased significantly compared to the baseline signal (*p*-value < 0.0001, which is statistically significant—see Fig. 6b), indicating the sensing of dopamine at its lower concentration (1 pm). With the spiked-plasma dilution, the sensor showed a deviation of ±3.8 µA from the mean signal (88.8 µA), which is ~ ±4%. Though the concentration of HP is high, the other neurotransmitters or proteins in plasma do not affect the dopamine sensing signal. This may be due to the presence of Nafion on the sensor surface which repels negatively charged proteins in plasma. Supplementary Fig. 10a, b shows the sensing data similar to that in Fig. 6a, b, respectively, but for AS where we observed results similar to that for the HP. These results indicate the clinical relevance of the 3D biosensing platform proposed in this work.

### Selectivity, reproducibility, and repeatability tests for the multi-length-scale sensing platform

**Fig. 7:**
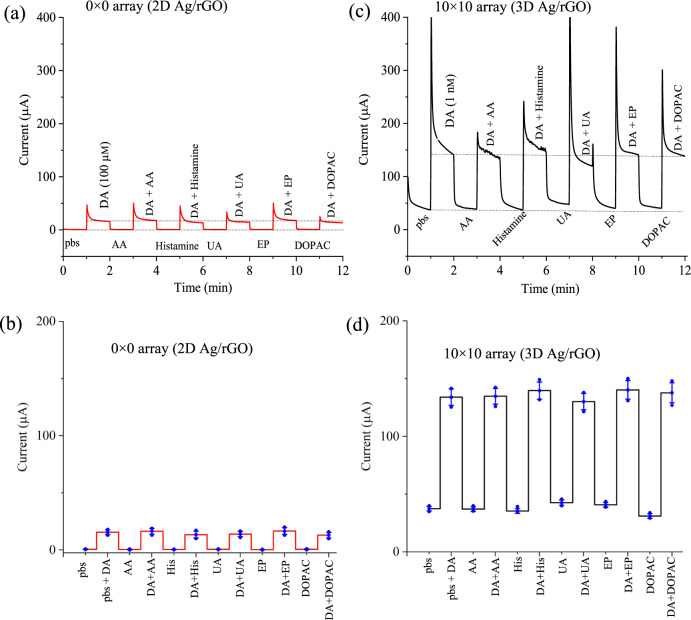
Selectivity tests for dopamine sensors. Current as a function of time for (**a**) 2D Ag/rGO sensor and (**c**) 3D Ag/rGO sensor with and without dopamine by introducing a variety of interfering neurotransmitters such as ascorbic acid (AA), histamine (His), uric acid (UA), epinephrine (EP) and 3,4-Dihydroxyphenylacetic acid (DOPAC) at a potential of −0.1 V. Current responses are plotted against interferents for 2D Ag/rGO (**b**) and 3D Ag/rGO (**d**). Error bars in (**b**) and (**d**) are the SD of measurements over at least *n* = 3 biologically independent experiments. Error bars, mean ± SD.

The selectivity test of the sensors was conducted using chronoamperometric method with and without dopamine in presence of a variety of interferents such as ascorbic acid (AA), histamine (His), uric acid (UA), epinephrine (EP), and 3,4-Dihydroxyphenylacetic acid (DOPAC) at a potential of −0.1 V (Fig. 7a, b). For these tests, the concentration of AA, His, UA, EP, and DOPAC was set to 10 μm. For this test, we used two different concentrations of dopamine, 100 µm for 2D Ag/rGO sensor and 1 nm for 3D Ag/rGO sensor. The 2D Ag/rGO sensor (Fig. 7a, b) exhibited a RSD of ±7.3% compared to the base signal and a RSD of ±6.4% compared to the signal with 100 µm concentration of dopamine in presence of other neurotransmitters. The 3D Ag/rGO sensor (Fig. 7c, d) exhibited a RSD of ±6.6% compared to the base signal and a RSD of ±3.4% compared to the signal with 1 nm concentration of dopamine in presence of other neurotransmitters. The oxidation potential (−0.14 V) determines the selectivity where the large area of 3D electrode structure allows a catalytic reaction only for the dopamine molecules. A low RSD of ±3.4% of 3D Ag/rGO sensor indicates the high specificity with the interfering molecules compared to the 2D Ag/rGO sensor (RSD~ ±6.4%). Thus, compared to the 2D sensor, the AJ printed 3D Ag/rGO micropillar electrodes provide a high selectivity for dopamine against other similar interfering neurotransmitters.

**Fig. 8:**
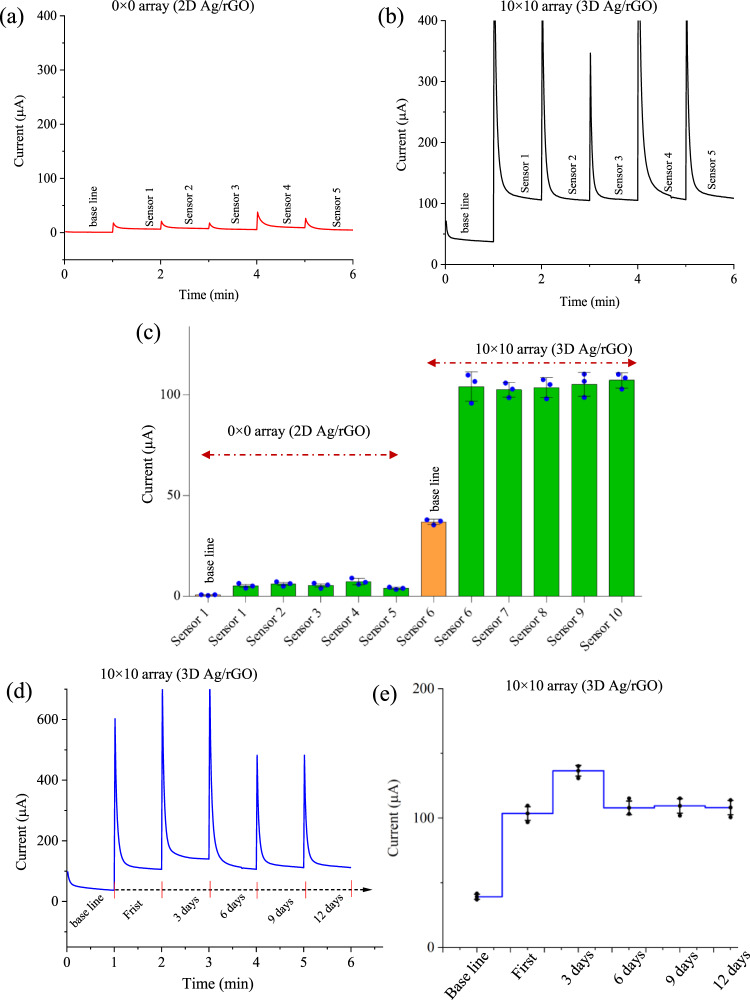
Reproducibility and stability tests of the multi-length-scale sensing platform. Five different sensors for each configuration (2D Ag/rGO and 3D Ag/rGO) were fabricated using the process developed in this work and tested with the same parameters in pbs solution with 1 mm of ferro/ferricyanide for 2D Ag/rGO electrode (**a**) and 3D Ag/rGO micropillar array electrode (**b**). The dopamine concentration was set to 20 µm for 2D Ag/rGO sensors and 1 pm for 3D Ag/rGO sensors. **c** The plot between chronoamperometric currents and the number of sensors for both 2D Ag/rGO and 3D Ag/rGO sensors. **d**–**e** The stability test of 3D Ag/rGO sensor was conducted at 1 pm concentration of dopamine by varying time in days while the sensor was kept at 4 °C. Error bars in (**c**) and (**e**) are the SD of measurements over at least *n* = 3 biologically independent experiments. Error bars, mean ± SD.

We also carried out sensor reproducibility tests with ten sensors—five identical sensors of 2D Ag/rGO (Fig. 8a) and five identical sensors of 3D Ag/rGO (Fig. 8b). The tests were conducted at a fixed dopamine concentrations of 20 μm and 1 pm at −0.14 V for 2D Ag/rGO and 3D Ag/rGO sensors, respectively. The high uniformity of 3D micropillar geometry (Supplementary Fig. 2) leads to a high reproducibility of the sensor (Fig. 8a–c) as evidenced by its low RSD value of ±0.61% compared to the 2D Ag sensor (RSD ~±12.1%). In comparison to the lithographically manufactured sensor for breast cancer biomarkers^37^, this result indicates a reasonable reproducibility and reliability for the multi-length-scale dopamine sensor.

Our sensor also showed a suitable stability of detection over at least 12 days of measurements when stored at 4 °C while not in use (Fig. 8d, e). The 3D Ag/rGO sensor exhibited a reasonable stability over time as evidenced by its low RSD of ±6.2% compared to its initial signal. The good stability of the sensor is likely because we do not incorporate any enzyme molecules at the electrode surface. The slight reduction of the current may, however, be due to the existing carbon in the base silver micropillars which may bind to oxygen from the buffer solution during electrochemical tests. To examine this, we have further conducted X-ray photoelectron spectroscopy (XPS) to investigate the surface elements present on the silver micropillars.

### Surface chemistry of 3D microelectrodes

For the multi-length-scale sensing platform developed in this work, we carried out XPS studies on the electrodes as the base electrode is made of sintered silver nanoparticles and we wanted to investigate its surface oxidation. Supplementary Fig. 11 shows the representative XPS spectra for an as-fabricated silver micropillar and that exposed to dopamine sensing without rGO coating, including (a) Ag 3*d*, (b) C 1*s*, (c) O 1*s*, (d) Na 1s, (e) *S* 2*p*, and (f) *P* 2*p* peaks. Peak intensities have been rescaled to show additional details, while quantified results are shown in Supplementary Table 3. It is clear that the sodium content in both samples is similar (~8%), which likely originated from the reducing agent during fabrication of the silver micropillars. After exposure to dopamine, more carbon and oxygen species are found on the surface of 3D silver electrodes, which block the signal of silver. No new oxygen (Supplementary Fig. 11a) or carbon (Supplementary Fig. 11c) species can be identified from the peak binding energies in XPS after sensing. Although more oxygen was present after sensing, the peak positions of Ag 3*d*_3/2_ (374.3 eV) and Ag 3*d*_5/2_ (368.3 eV) did not shift and no new peaks appeared at higher binding energies, meaning that the oxidation state of Ag did not change after dopamine sensing (i.e., no significant oxidation occurred). Interestingly, a trace amount of sulfur was detected in as-fabricated micropillars but disappeared after dopamine sensing (Supplementary Fig. 11d). Further, minor phosphate (~6%) was present after dopamine sensing (Supplementary Fig. 11e), which likely originated from pbs solution. As demonstrated in the Ag 3*d* spectra (Supplementary Fig. 11a), either no peak appeared due to oxygen or no changes of silver peak position occurred—which indicates that the darkening of the microelectrodes could be due to the existence of carbon residue in 3D silver that binds to oxygen species during oxidation of water molecules in pbs under a low potential. The change in color did not affect the oxidation of dopamine molecules and the sensing results shown in this paper.

The fabrication and testing of multi-length-scale micropillar-based sensing platform fabricated by AJ nanoparticle printing method represents a significant advance in the development of biosensing devices. In addition to demonstrating the sensing of dopamine at the lowest LoD yet reported in literature, this paper illustrates several novelties as discussed below.

The significant improvement in the LoD for the multi-scale 3D structure shown in Fig. 4 cannot be explained by the increase in the surface area alone for our sensor architecture. While the coating by rGO sheets is expected to enhance the surface area of the multi-length-scale architecture significantly, a proportional increase also occurs for the 2D surfaces coated with the rGO sheets. The micropillars, however, create a multi-length-scale architecture, where the rGO nanosheets increase the electrode surface area, while the hollow micropillars create a secondary structure that can interact with the analyte molecules even at a very low analyte concentrations in reasonable amount of time (all the test results in Fig. 4 were obtained within 60 s). The effect of multi-length-scale architecture on the enhancement of sensitivity for detection of biomolecules was predicted by Sheehan and Whitman^1^ and elaborated thereafter^7^. We also note that the sensor we developed in this work is demonstrated for in vitro detection of dopamine. However, recent developments on freestanding carbon sensors^51^ used in vivo for the detection of dopamine can provide a pathway to extend our work for in vivo applications, which will be part of a future investigation.

We note that AJ 3D printing offers distinct advantages for our platform compared to sensors made by lithographic methods. The hollow ring-shaped surface features and the sintered nanoparticle texture (Fig. 2) of the micropillar surfaces can enhance the coupling between the rGO sheets and the micropillar. The 3D printed architecture, designed via simulations (e.g. Fig. 2), can provide guidelines to design similar electrode geometries for a variety of biosensing applications. More complex architectures such as micropillars with higher aspect ratios or microlattices can also be used to further enhance this effect^19^. From a manufacturing perspective, the AJ printing of the electrode can be accomplished via simple CAD programs, followed by a batch process of oven sintering. This leads to a simple 2-step fabrication process that can be carried out at remote locations without the need for access to an expensive infrastructure associated with cleanrooms. This simplicity in the manufacturing process will lower the cost and ‘democratize’ biosensing since fabrication and testing can be carried out as needed at remote underserved populations. The idea of 3D printing of biosensors also enables a decentralized and scalable manufacturing set-up, which might be needed in situations such as pandemics.

We note that the AJ printing process used in this paper is based on droplet dynamics rather than the type of nanoparticles used^19,54,55^. Other materials such as gold nanoparticles have also been used to create the 3D architectures^56^. This flexibility in using different materials is unlike the chemical compatibility required in lithographic processes and can lead to several types of high-performance sensors. In addition, the availability of a wide range of electrode materials can avoid the requirements of redox mediators and enzyme molecules (other than affinity-based sensing) that addresses issues such as biofouling. For example, in this paper, the use of silver allows direct oxidation of dopamine due to its electro-catalytic properties. Further, it is noted that 3D printing can create structures that have imperfections larger than conventional lithography. For example, the micropillar structures used in the current work (Fig. 2) have a jagged surface that is a result of the AJ printing and nanoparticle sintering. This feature of 3D printing, however, can be used as an advantage in electrochemical processes with the jagged texture increasing the surface roughness and porosity, which can enhance electrochemical reactions. Finally, the availability of a device that can detect dopamine at femtomolar sensitivity will open new research areas in neuroscience and in neurodegenerative diseases, as the detection at these (low) concentrations were simply not possible in the past. The flexibility of 3D printing can also be utilized to create implantable microneedles for the detection of dopamine or other neurotransmitters which will have significant clinical relevance and will be part of a future investigation. Overall, the research presented in this paper opens up the exciting possibility of using nanoparticle 3D printing to create a rapid multi-length-scale sensing platform that detects biomolecules down to femtomolar (or even lower) concentrations and benefit the areas of disease diagnostics and its prevention and treatment.

In summary, we have developed a rapidly manufactured high-performance multi-length-scale sensing platform for biomolecules and demonstrated that it can be used to detect dopamine, an important neurotransmitter, at femtomolar concentrations. The sensor consists of micro- and mesoscale structures of hollow micropillar array electrodes fabricated by AJ nanoparticle 3D printing, and a surface coating of rGO nanoflakes. The electrode was integrated with a microfluidic device to form the sensor. Electrochemical simulations demonstrated that the 3D electrode structure introduces both the linear and radial diffusion of ions due to their geometry during dopamine detection and were used to design the appropriate array configuration applied in the experiments. The excellent electro-catalytic properties of silver nanoparticles and a larger surface area with multi-length-scale hierarchical structure of the 3D geometry coupled with rGO allowed quantification of dopamine at femtomolar concentration. In addition, with high redox activity, the sintered nanoparticles in the 3D electrode provided direct electron transfer from electrolyte to current collector with high diffusion co-efficient, and a high HET rate compared to a traditional 2D sensor geometry. Lastly, the sensor platform showed rapid detection (60 s), high sensitivity, and in vitro capability of sensing dopamine in spiked human plasma and serum samples. This work, in addition to being an important advance in biosensing enabled by advances in microelectronics fabrication, enables the detection of dopamine at hitherto undetectable ranges, which will open new pathways for the detection and treatment of neurodegenerative and other diseases.

## Methods

### Materials

To construct the 3D hollow micropillar array, we used silver nanoparticle ink (Prelect TPS 50 G2, Clariant Group, Frankfurt, Germany). The size of the nanoparticles in the ink was 30–50 nm, the ink viscosity was about 1.5 cP, and the silver particle loading in the ink was about 40 ± 2 wt%. Silver was chosen because of the excellent electro-catalytic properties exhibited by silver nanoparticles in oxidizing other biomolecules such as glucose^57^. The rGO nanoflakes in powder form were purchased (CAS‐No. 7782‐42‐5) from ACS Materials LLC, Pasadena, CA, USA. As per the manufacturer, the rGO was synthesized from graphene precursor via a reduction process of hydrazine (N_2_H_4_) treatment, and the rGO sheets has a thickness of ~1 nm, a conductivity of >500 Sm^−1^, and a diameter of 0.5–10 µm. The solvents used for this ink were deionized (DI) water and ethylene glycol. Ethylene glycol acted as a humectant which is known to help in the formation of non-planar structures required for this work^19^. Fetal bovine serum (fbs) and rabbit serum (rs) were obtained from Sigma Aldrich (St. Louis, MO, USA). Polydimethylsiloxane or PDMS (SYLGARD™ 184 Silicone Elastomer Kit, Dow Corning, Midland, MI, USA) was used as the housing material to form the microfluidic channel in the sensor device by mixing the base to hardener at a ratio of 10:1 using a process described in the next section. Artificial serum (SMx Serum) was purchased from UTAK, Inc., Valencia, CA, and HP (product number: P9523) was obtained from Sigma Aldrich (St. Louis, MO). The experiments with biological fluids were conducted in the biosafety cabinet level 2 (BSL 2) facility in the biomedical engineering department at Carnegie Mellon University.

### Fabrication of micropillar array electrodes

Figure 1 shows schematics of the sensor device and the fabrication process of the 3D micropillar sensor array electrodes using AJ nanoparticle printing. The process consists of patterning a 100 nm thick Au layer on a glass substrate using an e-beam evaporator (Kurt Lesker PVD 75, Jefferson Hills, PA USA) followed by the deposition of a 5 nm thick chromium (Cr) adhesion layer using the same method via a pre-cut shadow mask of Kapton^®^ tape (Silhouette America^®^, Inc.). This pattern was used to create three electrodes, namely, WE, CE, and RE. The AJ printing process was then used to print 3D micropillar array (10 × 10 hollow pillars over an area of 2 mm × 2 mm) on the surface of the WE (Fig. 1c, g).

The Aerosol Jet 3D printer (Model AJ-300, Optomec, Inc., Albuquerque, NM USA) used in this work had three atomizers (two ultrasonic atomizers and one pneumatic atomizer), a programmable heated X-Y motion stage (i.e. platen), and a deposition head. For the construction of the microelectrode array in this work, an ultrasonic atomizer was used to aerosolize the silver nanoparticle ink as shown in Fig. 1a. The aerosolized ink droplets were carried to a nozzle by a carrier gas (N_2_). Inside the nozzle, the droplets were focused towards the nozzle tip with the help of a sheath gas (also N_2_) to form a micro-jet. The process of printing was carried out by a continuous flow of droplets which was diverted or resumed by the movement of a shutter (not shown). The diameter of the nozzle tip was 150 μm, which is known to give rise to an aerosol stream having a diameter of ~10–15 μm. Before printing, the geometry of the conductive part was drawn in AutoCAD, 2020 using a program in the software AutoLISP (AutoCAD 2020, Autodesk Inc., San Rafael, CA) and converted to a ‘prg’ file compatible with the printer software. The desired shape of an individual hollow micropillar is shown in Fig. 1d, g. The carrier and sheath gas flow rates during printing (Fig. 1d, e) were 25 sccm and 50 sccm, respectively. The dried micropillar structure was then heated to 320 °C for 2 h to remove the binder and sinter the nanoparticles. For comparison with 3D sensor structures, several 2D silver block electrodes with similar overall dimensions (2 mm × 2 mm × 0.05 mm) were fabricated using AJ printing method on WE and also used to detect dopamine.

### Fabrication of microfluidic sensor device

Figure 1f shows the construction of the microfluidic device for dopamine sensing. The device consists of three electrodes (CE, RE, and WE) on glass substrate and a PDMS housing that forms the microfluidic channel. The CE consists of the as-deposited Au and Cr layers. The RE was formed by depositing a Ag/AgCl layer on top of the Au/Cr layer. To achieve this, a 2 mm × 1 mm area of the RE (i.e., part of the RE under the PDMS housing in the microfluidic channel as shown in the schematic in Fig. 1g) was coated with commercially available Ag/AgCl material (Ag/AgCl paste, Ercon Inc., Wareham, MA USA) via a shadow mask-assisted screen printing. The RE was then annealed at 150 °C for 2 h to remove the solvents. The WE consisted of the AJ printed micropillar array on top of the Au/Cr layer as shown in Fig. 1c, f. The silver microelectrode array was functionalized with rGO and 1% Nafion.

The rGO sheets were dispersed in deionized water (1 mg mL^−1^), added with Nafion (5.0%) solution (obtained from Sigma Aldrich, St. Louis, MO, USA) and sonicated for 1 h. A PDMS fence was created that surrounded the silver microelectrode arrays, and a 20 µL solution of the mixture of rGO and Nafion was drop-casted onto the array surface and dried at 85 °C for 2 h. This process was repeated three times to ensure a uniform coverage by rGO flakes of the microelectrodes array. Similarly, other electrodes such as 2D (0 × 0 array) and 3D (4 × 4 array) were functionalized with rGO flakes and Nafion. Adding the perfluorinated polymer layer of Nafion onto microelectrode array can act as a barrier and repels the negatively charged neurotransmitters such as ascorbic acid (and other negatively charged neurotransmitters) but allows the interaction with positively charged molecules such as dopamine^58^, thereby providing high selectivity. Note that based on the 3D construction of the electrode alone, the area increases for the micropillar structures over a planar electrode (Fig. 1f) is about 260%. The approximate dimensions of the micropillars as programmed in the 3D printer are given in Fig. 1g. The rGO-coated silver micropillar arrays depicted in Figs. 1f and 2a–c act as the selective and sensitive electrodes for dopamine detection without the need for any enzyme molecules.

The PDMS housing that contained the electrodes and the microfluidic channel shown in Figs. 1f and 2a was fabricated by a replica molding method depicted in Supplementary Fig. 1. First, a mold was created via milling of a 1/2-inch-thick polymethylmethacrylate (PMMA) slab (step-1 of Supplementary Fig. 1a, b). This mold had the replica of a microfluidic channel having a depth and length of 1 and 20 mm, respectively, and width of 1 and 2 mm (Supplementary Fig. 1c). A PDMS hardener and base mixture was prepared and degassed in a vacuum chamber for 30 min at 10^−4^ Torr and then poured into the PMMA mold. The PDMS was cured inside the PMMA mold on a hot plate at 80 °C for 2 h (step-2 of Supplementary Fig. 1a, b). Once cured, the PDMS was peeled off from the mold. This created a master PDMS mold, with the opposite shape as that of the channel of interest. The master PDMS mold was then treated with silicone oil (Ease Release™ 205, Reynoldsam Advanced Materials, Macungie, PA USA) as shown in step-3 of Supplementary Fig. 1a, b. A new PDMS mixture was then poured on the master PDMS mold, cured at 80 °C for 2 h and then peeled off to create the PDMS housing used in the device (step-4 of Supplementary Fig. 1a, b). This housing was then placed on a glass substrate with the electrodes to complete the electrochemical cell circuit as shown in Fig. 1g. The inlet and outlet of the device were created by punching holes into the PDMS and using tygon tubes for the insertion of fluids into the cell. Successive injection of dopamine at different concentrations along with buffer/serum solutions was then carried out to detect the electrochemical signal. The electrodes were connected to an electrochemical workstation (VersaSTAT 3 Potentiostat Galvanostat, Princeton Applied Research, Oak Ridge, TN USA) to measure the readout signals.

### Electrode characterization and electrochemical simulations

The micropillar array electrodes were characterized using scanning electron microscope or SEM (FEI Sirion SEM, Hillsboro, OR USA). The surface chemistry of the samples with silver (without rGO) before and after electrochemical measurements was assessed by XPS using a Thetaprobe (ThermoFisher, Waltham, MA). The XPS characterization was performed at room temperature with a base pressure of 3 × 10^−9^ mbar. Samples were mounted on a glass substrate, and charge compensation was achieved by using a dual-mode flood-gun with a mixture of −2 eV electron and Ar^+^ as neutralization source. Al K_-α_ X-ray source of 1486.8 eV energy was used with an oval spot (400 × 600 μm^2^ in size) projected to sample surface. Further, 150 eV pass energy and 400 ms dwell time were used for the XPS measurement. The signal was detected from top of the pillars of the array.

Simulations were carried out to investigate the diffusion profiles and the total current inside the microfluidic chamber during the oxidation/reduction of dopamine for different micropillar array configurations using COMSOL Multiphysics software (Version 5.5, COMSOL Inc., Burlington, MA USA). In the electroanalysis module of COMSOL, electrochemical reaction was used for a single species (B) and formed product (A) via oxidation (B + ↔A + e^−^). At the electrode boundary, the species ‘B’ oxidizes to ‘A’ wherein the bulk concentration is uniform, and the product concentration is zero. The domain equation in this simulation was the diffusion equation of Fick’s second law. To evaluate the total current of the micropillar arrays, we used the electroanalytical Butler-Volmer equation^59^ for the oxidation reaction. In this simulation, a total of eight array configurations, namely, 0 × 0, 1 × 1, 2 × 2, 4 × 4, 6 × 6, 8 × × 8, 10 × 10, 12 × 12, 14 × 14, and 20 × 20, were analyzed. The diffusion profiles of the species at the electrode and the total current were calculated to obtain an optimum array configuration for the sensor.

### Software

For statistical analysis, the OriginPro 2020 and GraphPad Prism 6.02 were used. Solidworks 2019 was used to create 3D schematics. AutoCAD 2020 was used to create circular patterns for 3D printing. The software associated with the Aerosol Jet 3D printer is the Kewa Gadget, wherein different control managers, namely, motion manager 3.2.83, process control 3.2.83, and vision manager 3.2.83 were used to control the 3D printing of nanoparticles.

### Reporting summary

Further information on research design is available in the Nature Research Reporting Summary linked to this article.

## Supplementary information

Supplementary Information

Reporting Summary

## Data Availability

All relevant data that support the findings of this study are presented in the manuscript and supplementary information file. Source data are available from the corresponding author upon reasonable request.
