# Supplementary material for: Breaking the barrier to biomolecule limit-of-detection via 3D printed multi-length-scale graphene-coated electrodes
Source: Nat Commun. 2021 Dec 6;12:7077. doi: 10.1038/s41467-021-27361-x (PMC8648898; doi:10.1038/s41467-021-27361-x)
Supplement: Supplementary file 1 — Supplementary Information [file 41467_2021_27361_MOESM1_ESM.pdf]

Supplementary Information

Breaking the Barrier to Biomolecule Limit-of-Detection via 3D Printed Multi-length-scale Graphene-coated Electrodes

Md. Azahar Ali[1], Chunshan Hu[1], Bin Yuan[1], Sanjida Jahan[1], Mohammad S. Saleh[1], Zhitao Guo[2], Andrew Gellman[2], and Rahul Panat[1,*]

[1]*Department of Mechanical Engineering, Carnegie Mellon University, Pittsburgh, PA 15213 USA*

[2]*Department of Chemical Engineering, and Wilton E. Scott Institute for Energy Innovation, Carnegie Mellon University, Pittsburgh, PA 15213 USA*

Supplementary Section 1: Calculation of Limit-of-Detection for Ag/rGO Sensor

The limit-of-detection (LoD) was evaluated using a method reported in literature[1].

Limit of blank (LoB) = Mean of signal (blank sample) + 1.645 × (Std dev of blank sample) (1)

Limit of detection of the signal (Y_{LoD}) = LoB + 1.645 × (Std dev of target at low concentration) (2)

LoD = (Y_{LoD} - C)/slope of the sensor calibration (3)

Here, '*C*' is the intercept of the calibration curve of the sensor.

For 2D Ag/rGO (0×0 array) sensor, the mean of the blank signal and standard deviation are 0.508 µA and 0.221 µA [*n*, replicate=3], respectively. From Eq. (1), the LoB is calculated as 0.871 µA. Mean signal at the lowest concentration (1 µM of dopamine) is 1.55 µA, with a standard deviation of 0.395 µA [*n*, replicate= 3]. This provides Y_{LoD} = 3.42 µA as per Eq. (2). The sensor calibration equation is Y_{LoD} (µA) = 17.95 × Log [*X* (µM)] – 15.5. This gives C = -15.5 and slope of the sensor calibration curve as 17.95. Plugging into Eq. (3), the LoD for 2D Ag/rGO (0×0 array) sensor is 11.2 µM.

For 3D Ag/rGO (4×4 array) sensor, the mean of the blank signal and standard deviation are 16.8 µA and 1.31 µA [*n*= 3], respectively. From Eq. (1), LoB is calculated as 18.95 µA. Mean signal at the lowest concentration (1 pM of dopamine) is 23.03 µA, with a standard deviation of 1.63 µA [*n*= 3]. This provides Y_{LoD} = 21.6 µA as per Eq. (2). The sensor calibration equation is Y_{LoD} (µA) = 2.42 × Log [*X* (µM)] + 36.5. This gives C = 36.5 and slope of the sensor calibration curve as 12.66. Plugging into Eq. (3), the LoD for 3D Ag/rGO (4×4 array) sensor is 0.87 pM.

For 3D Ag/rGO (10×10 array) sensor, the mean of the blank signal and the standard deviation are 38.3 µA and 1.69 µA [*n*= 3], respectively. From Eq. (1), the LoB is calculated as 41.08 µA. Mean signal at the lowest concentration (10 fM of dopamine) is 50.5 µA, with a standard deviation of 1.22 µA [*n*= 3]. This provides Y_{LoD} = 43.14 µA as per Eq. (2). The sensor calibration equation is Y_{LoD} (µA) = 12.66 × Log [*X* (µM)] + 173.6. This gives C = 173.6 and slope of the sensor calibration curve as 12.66. Plugging into Eq. (3), the LoD for 3D Ag/rGO (10×10 array) sensor is 0.5 fM.

Supplementary Table 1. The physical concentration ranges of dopamine in human biofluids.

Level	1 fM	1 pM; Plasmonic Sensors	1 nM; ELISA, HPLC, Flometry Electrochemical	1 µM; ELISA, HPLC, Electrochemical, Plasmonic
Whole Blood	No sensors	< 65pM (Resting adults)	0.01– 0.48 nM (adult)	
Plasma			< 0.13 nM (ambulatory adult)<br>< 0.39nM (3-9 years)	
Urine			523-2472 nM (3-8 years)<br>334-3100 nM (9-12 years)	0.332–4.2 µM (13-17 years)<br>0.34–3.1 µM (>17 years)

Supplementary Table 2. The electrochemical parameters such as diffusion co-efficient (D_0), surface area, and charge transfer resistance (R_{ct}) of the fabricated sensors.

Electrodes	Area (cm^2)	Oxi/Red Current	D_0 (Oxi) (μ cm^2/s)	D_r (Red) (μ cm^2/s)	Peak-to-peak Separation Voltage (V)	R_{ct} (Ω)
3D (10×10) Sensor	0.104	16.4/19.6	0.698	0.994	0.002	267
2D (0×0) Sensor	0.04	9.63/10.96	0.16	0.208	0.222	887

Supplementary Table 3. Composition of as-fabricated silver micropillars and those after exposure to pbs/dopamine calculated form the XPS spectra shown in Supplementary Figure 11 by Avantage software. We note that due to unstable charge compensation over time as well as across sample surface, peak positions in XPS collected on sample after sensing had to be shifted by less than ± 0.7 eV to match with that before the experiment; while peak positions for the as-fabricated sample matched well with available database and did not need further adjustment.

Sensor condition	C wt%	Ag wt%	O wt%	Na wt%	S wt%	P wt%
As fabricated	13.06	59.07	16.73	8.64	2.5	0
After dopamine sensing	22.52	32.68	29.86	8.79	0	6.15

Supplementary Figure 1. Schematic and images of the fabrication process for PDMS housing *via* replica molding. (a) Step wise fabrication process of the four identical microfluidic channels by transferring patterns on PMMA into PDMS. (b) Optical photographs of PMMA channels and patterns transferred onto PDMS with microfluidic channels. (c) Photograph of microfluidic channels with their dimensions.

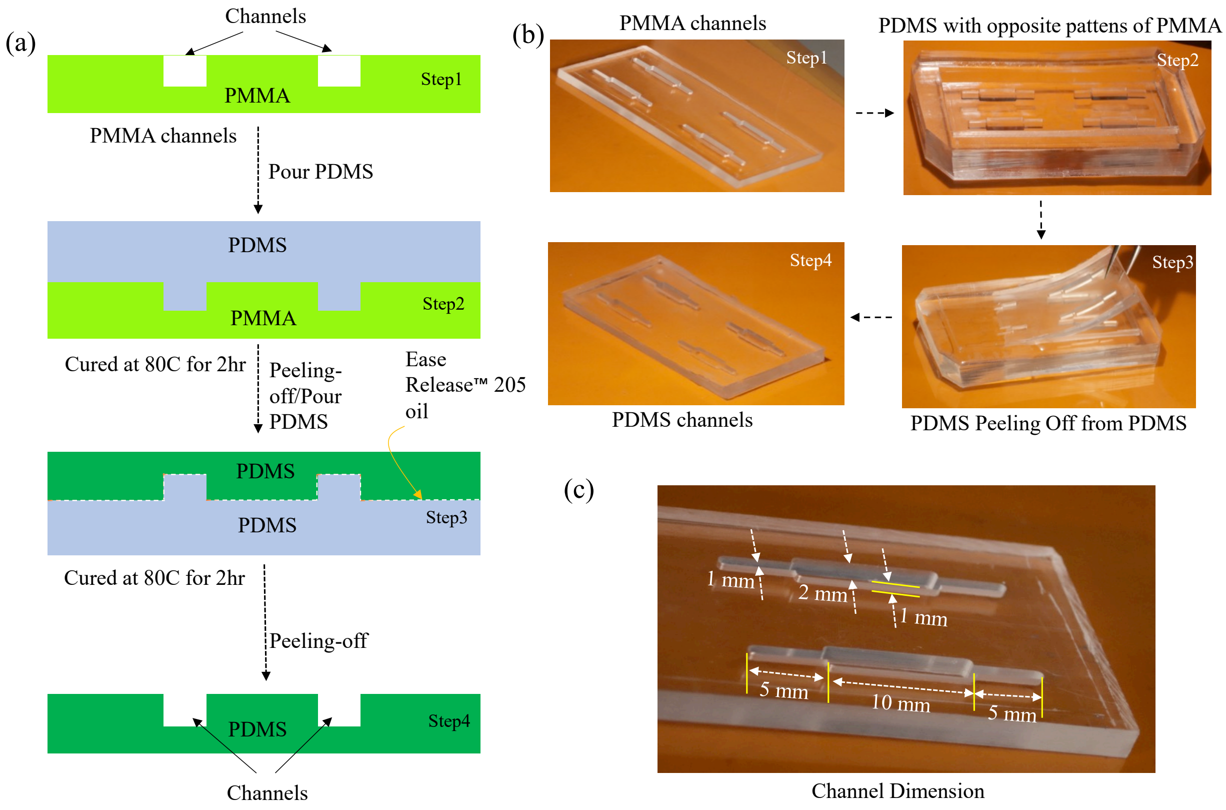

Supplementary Figure 2. Micropillar height (a), diameter (b) and pillar-to-pillar distance (c) of the fabricated sensors. Three sensors with six data points were measured to evaluate the height (a), diameter (b) and pillar-to-pillar distance (c). The error bars in (a), (b) and (c) correspond to the standard deviation of five (n = 5) measurements at randomly chosen micropillars in each sample. Error bars, s.e.m. ± SD.

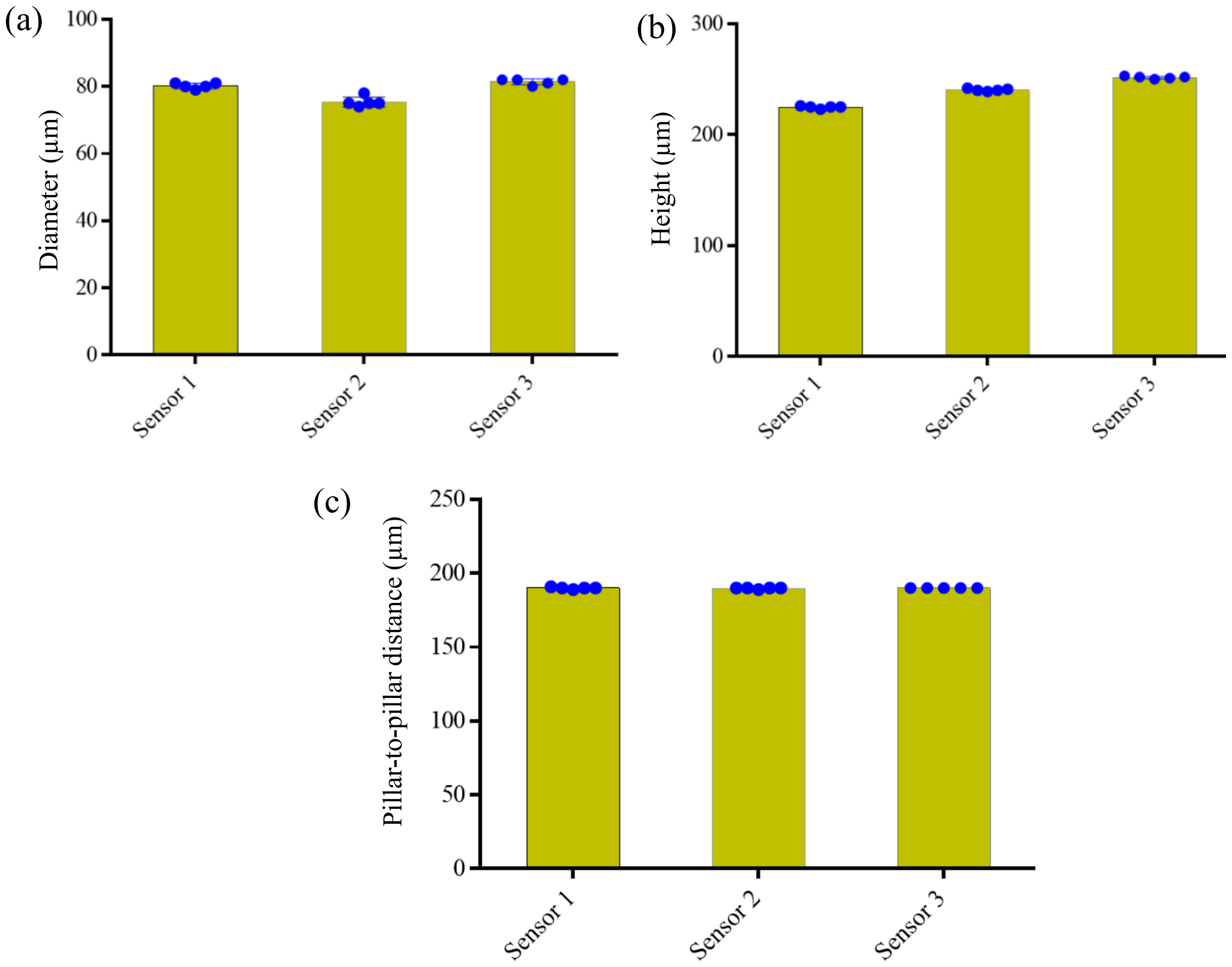

Supplementary Figure 3. Representative SEM image showing 4×4 array of silver micropillars (prior to coating with rGO). A total of 32 micropillars fabricated using AJ printing across two samples were imaged using SEM.

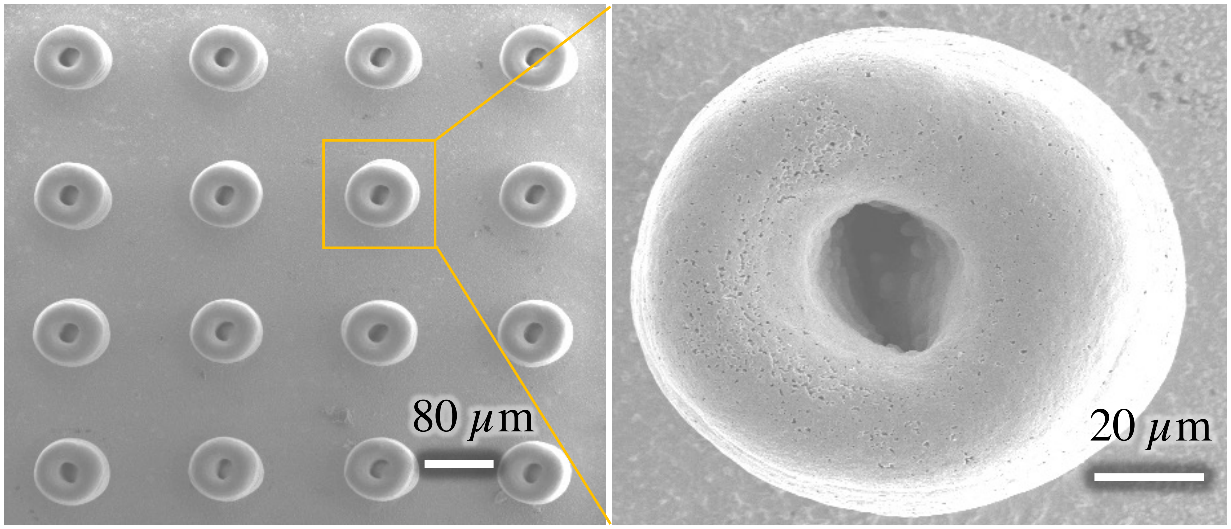

Supplementary Figure 4: (a-c) SEM images of AJ printed 2D silver electrode at various magnifications. A total of two 2D electrodes were observed by this technique.

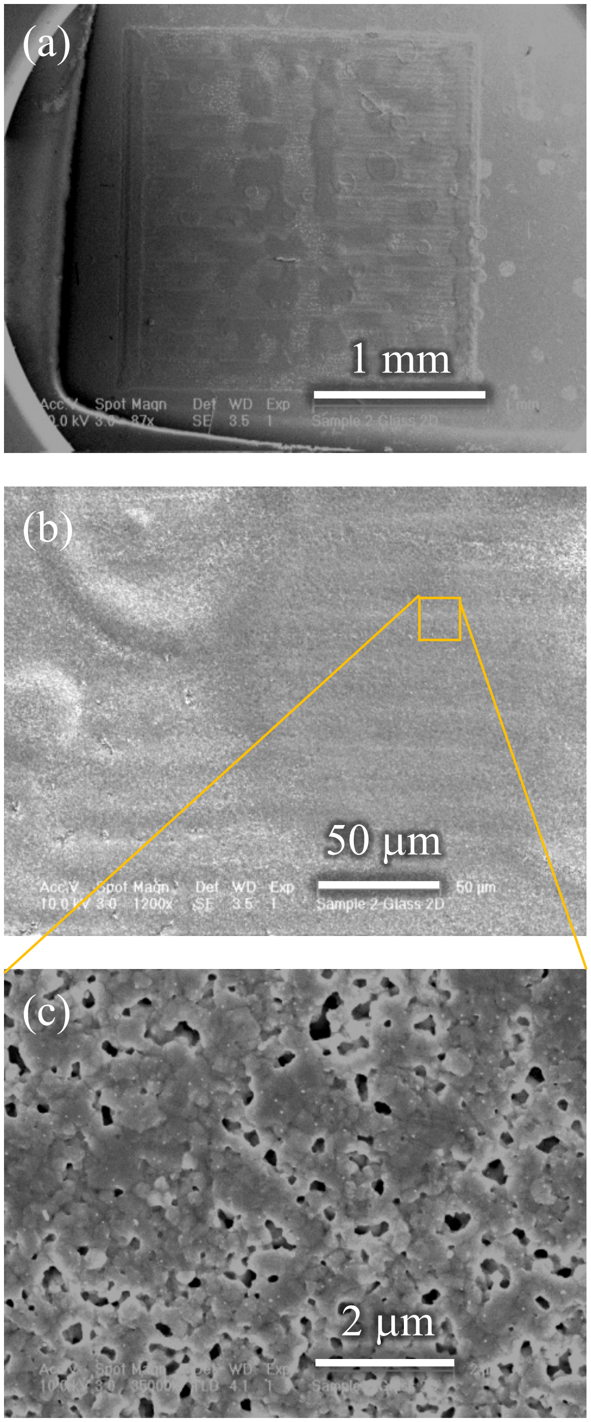

Supplementary Figure 5. COMSOL simulation for diffusion profiles of the electrode structures. Concentration of diffusion profile for 2D (0×0 array) and 3D (1×1 array) electrodes.

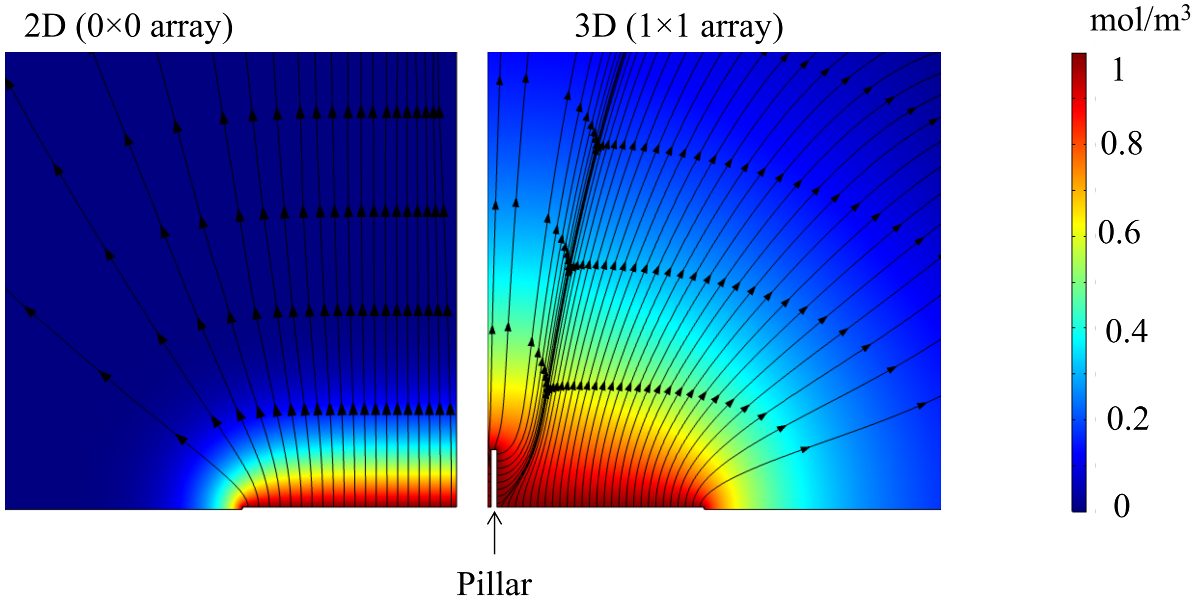

Supplementary Figure 6. Heterogeneous electron transfer rate constants (k_0) for different electrode configurations (error bars from n = 3, mean ± s.e.m.) at a scan rate of 0.05 V/s.

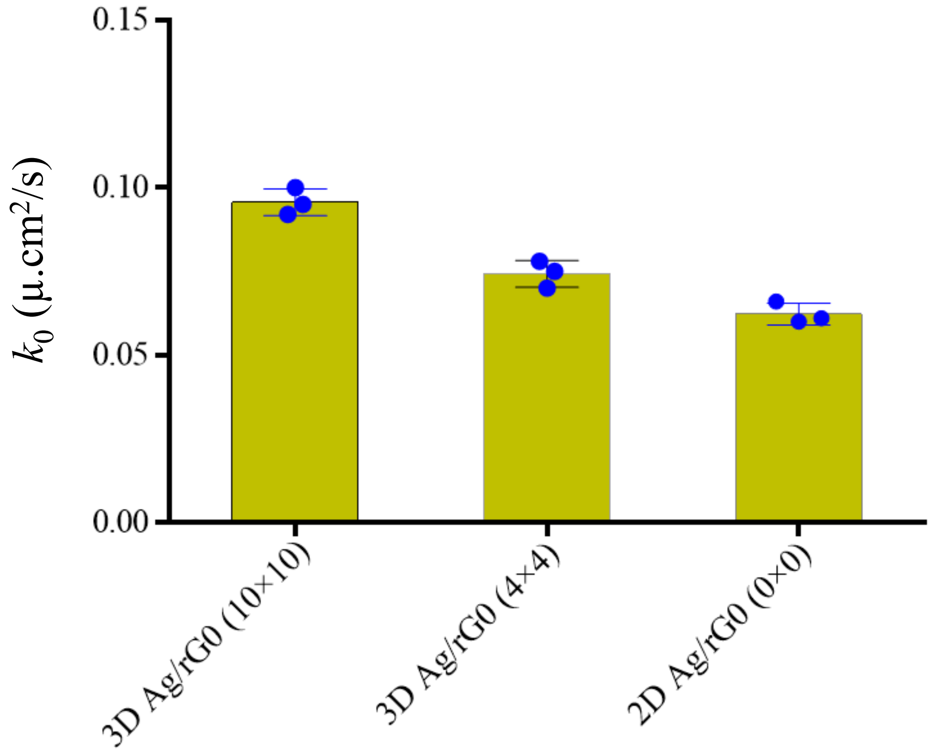

Supplementary Figure 7. The chronoamperometric response of three sensors with different configurations in pbs solution containing 1 mM of ferro/ferricyanide at constant oxidation potential (-0.14 V).

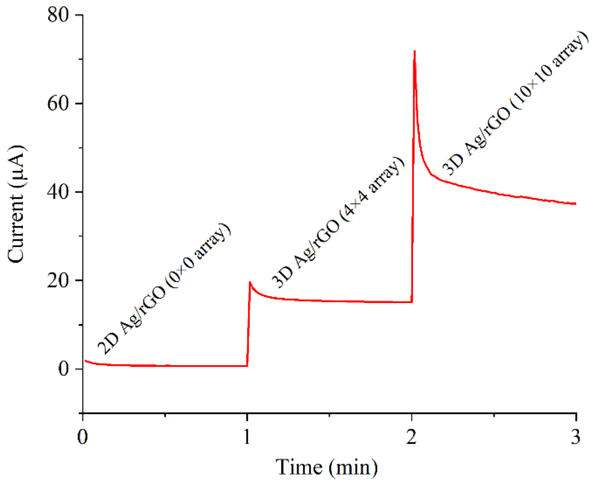

Supplementary Figure 8: **Plots showing current densities** for (a) planar 2D Ag/rGO sensor, (b) multi-length-scale 3D Ag/rGO (4×4 array), and (c) 3D Ag/rGO (10×10 array) sensors. These graphs are similar to Figure 4 (A, C, and E), but with the *y*-axis plotted as the current density. The concentration of dopamine was varied from 10 fM to 1000 mM in pbs solution (pH 7.4) containing 1 mM concentration of ferro/ferricyanide. The current density of each sensor is obtained by dividing their respective areas of the electrodes that are mentioned in the corresponding plot.

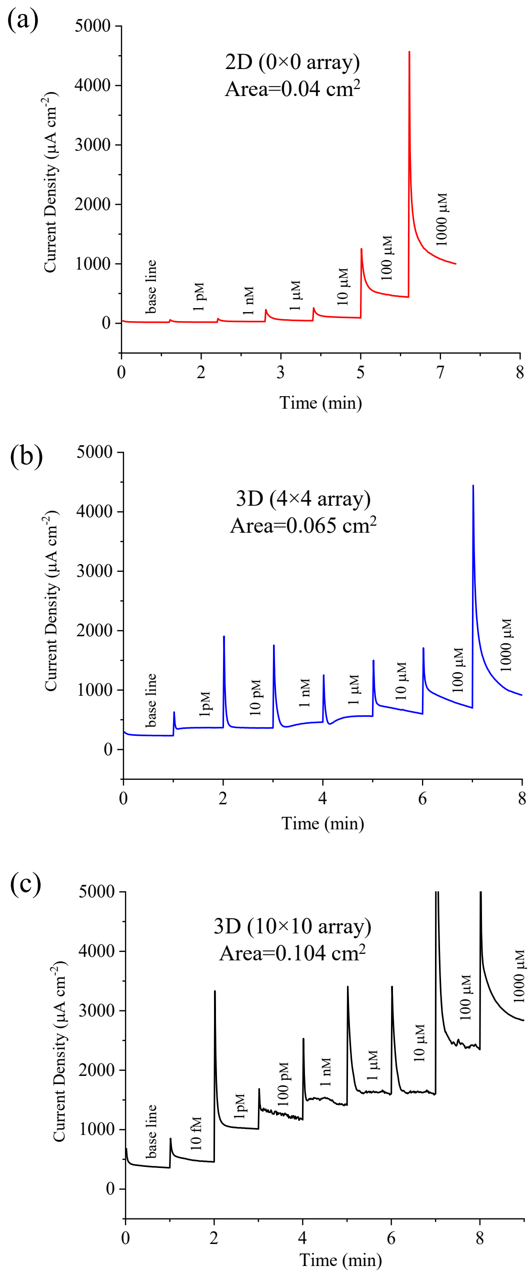

Supplementary Figure 9. Calibration Plots of Fabricated Dopamine Sensors. (a, b and c) Current responses with dopamine concentration for three different electrode configuration such as (a) 2D Ag/rGO (0×0 array), (b) 3D Ag/rGO (4×4 array) and (c) 3D Ag/rGO (10×10 array) electrodes. (d and e) Different visualization of the plots of the sensors with the overlay of current responses. Error bars in Figure (a-e) are the standard deviation of three repeated measurements (*n* = 3, biologically independent experiments). Error bars, median ± SD.

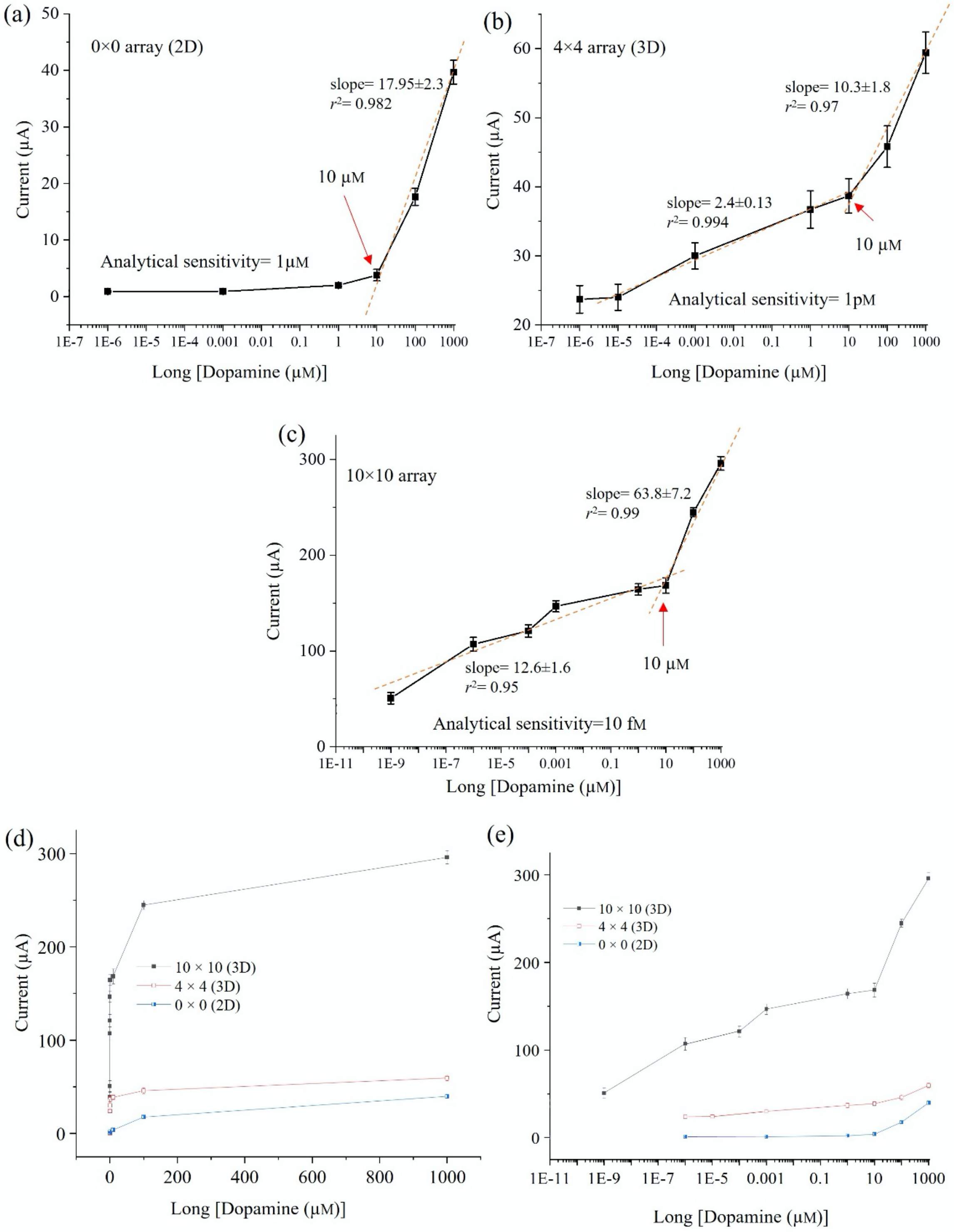
(a)
0×0 array (2D)
slope= 17.95±2.3
r^2= 0.982
10 μM
Analytical sensitivity= 1μM
Current (μA)
Long [Dopamine (μM)]
(b)
4×4 array (3D)
slope= 10.3±1.8
r^2= 0.97
slope= 2.4±0.13
r^2= 0.994
10 μM
Analytical sensitivity= 1pM
Current (μA)
Long [Dopamine (μM)]
(c)
10×10 array
slope= 63.8±7.2
r^2= 0.99
slope= 12.6±1.6
r^2= 0.95
10 μM
Analytical sensitivity=10 fM
Current (μA)
Long [Dopamine (μM)]
(d)
10 × 10 (3D)
4 × 4 (3D)
0 × 0 (2D)
Current (μA)
Long [Dopamine (μM)]
(e)
10 × 10 (3D)
4 × 4 (3D)
0 × 0 (2D)
Current (μA)
Long [Dopamine (μM)]

Supplementary Figure 10. 3D Sensor for the Detection of Dopamine in Artificial Serum (AS). (a) Response of the 3D printed sensing platform developed in this work for the detection of dopamine in artificial serum. Artificial serum was diluted in pbs solution in ratios of 1:5 to 1:500 (AS:pbs). The spiked artificial serum was made by adding a fixed dopamine concentration (1 pM) into all the diluted serum. The baseline measurement was carried out without adding any serum or dopamine. (b) Plot showing the current response (at 60 s) for the data in (a). Error bars in (b) are the standard error of the mean (s.e.m) of measurements over at least $n = 3$ biologically independent experiments. Error bars, mean ± s.e.m. Statistical significance was determined by one-way ANOVA test (non-parametric) where p value is 0.49 ($p = 0.49$) for artificial serum without dopamine and <0.0001 ($p < 0.0001$) for artificial serum with dopamine, indicating dopamine detection in artificial serum by the 3D sensor. p values for each comparison are labeled on top of the graphs.

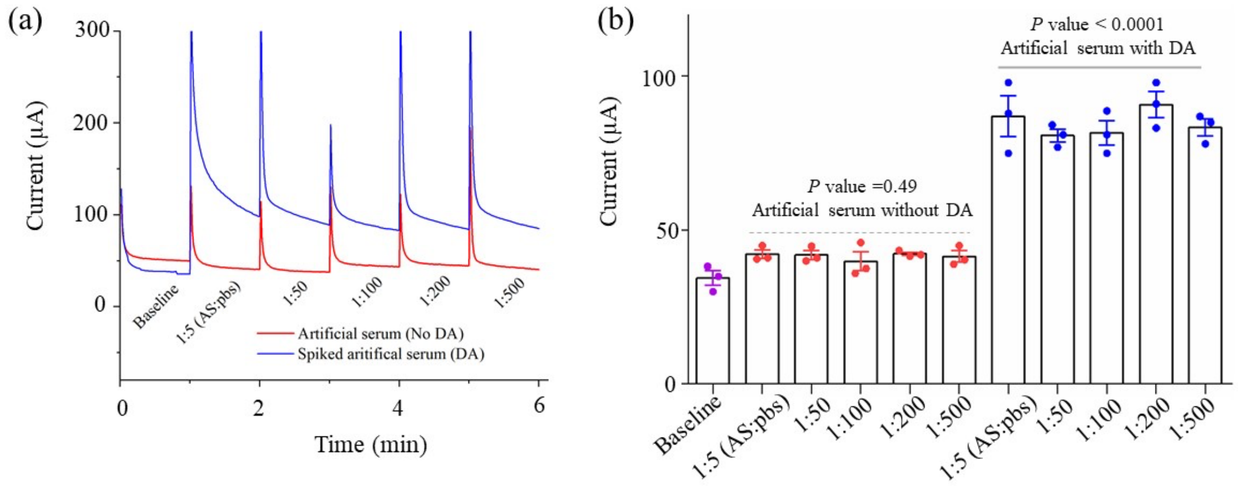

Supplementary Figure 11. XPS spectra of the 3D Ag/rGO micropillar sensor before and after the experiments of dopamine sensing. XPS spectrum of (a) Ag 3*d*, (b) O 1*s*, (c) C 1*s*, (d) Na 1*s*, (e) *S* 2*p*, and (f) *P* 2*p* on sensors before and after the exposure to pbs solution. Oxidation state of Ag remains unchanged after experiment. Intensities have been rescaled to help visualization, and quantitative results are shown in Supplementary Table 3.

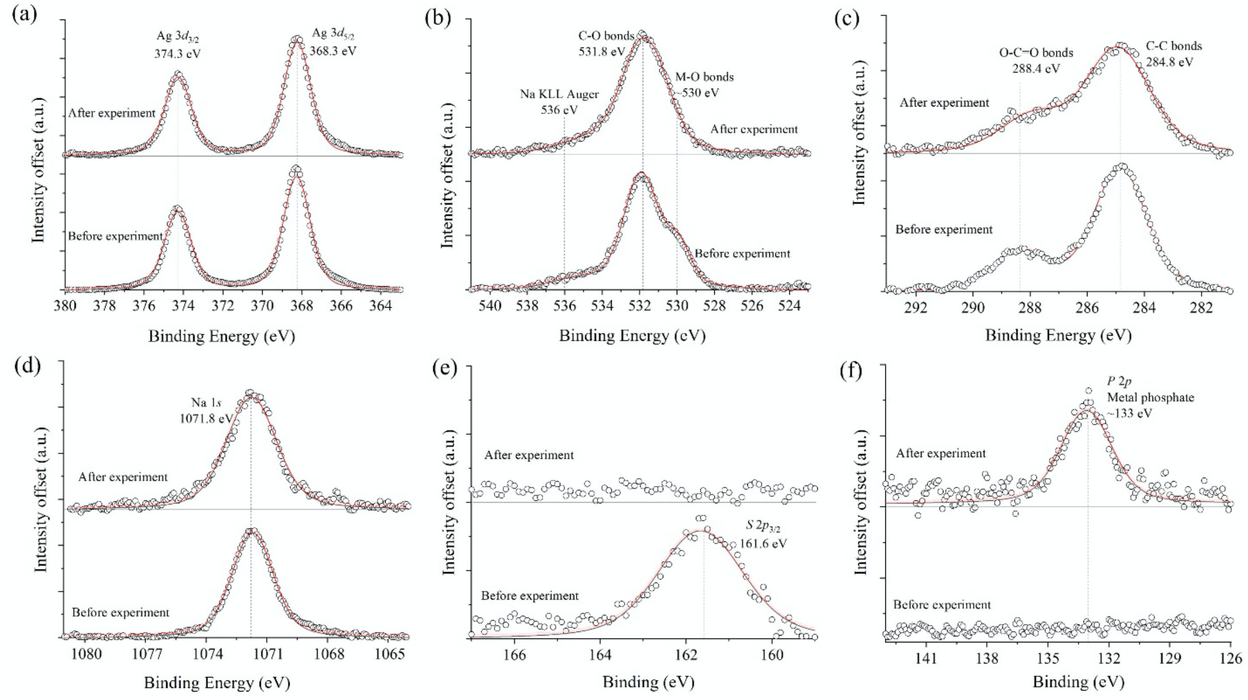

References

1 Lavín, Á. *et al.* On the determination of uncertainty and limit of detection in label-free biosensors. *Sensors* **18**, 2038 (2018).
