# Supplementary material for: Breaking the barrier to biomolecule limit-of-detection via 3D printed multi-length-scale graphene-coated electrodes
Source: Nat Commun. 2021 Dec 6;12:7077. doi: 10.1038/s41467-021-27361-x (PMC8648898; doi:10.1038/s41467-021-27361-x)
Supplement: Supplementary file 2 — Reporting Summary [file 41467_2021_27361_MOESM2_ESM.pdf]

nature portfolio

Corresponding author(s): Rahul Panat

Last updated by author(s): Oct 16, 2021

Reporting Summary

Nature Portfolio wishes to improve the reproducibility of the work that we publish. This form provides structure for consistency and transparency in reporting. For further information on Nature Portfolio policies, see our Editorial Policies and the Editorial Policy Checklist.

Statistics

For all statistical analyses, confirm that the following items are present in the figure legend, table legend, main text, or Methods section.

n/a	Confirmed	
☐	☒	The exact sample size (n) for each experimental group/condition, given as a discrete number and unit of measurement
☐	☒	A statement on whether measurements were taken from distinct samples or whether the same sample was measured repeatedly
☐	☒	The statistical test(s) used AND whether they are one- or two-sided<br>*Only common tests should be described solely by name; describe more complex techniques in the Methods section.*
☒	☐	A description of all covariates tested
☒	☐	A description of any assumptions or corrections, such as tests of normality and adjustment for multiple comparisons
☐	☒	A full description of the statistical parameters including central tendency (e.g. means) or other basic estimates (e.g. regression coefficient) AND variation (e.g. standard deviation) or associated estimates of uncertainty (e.g. confidence intervals)
☐	☒	For null hypothesis testing, the test statistic (e.g. F, t, r) with confidence intervals, effect sizes, degrees of freedom and P value noted<br>*Give P values as exact values whenever suitable.*
☒	☐	For Bayesian analysis, information on the choice of priors and Markov chain Monte Carlo settings
☒	☐	For hierarchical and complex designs, identification of the appropriate level for tests and full reporting of outcomes
☒	☐	Estimates of effect sizes (e.g. Cohen's d, Pearson's r), indicating how they were calculated

Our web collection on statistics for biologists contains articles on many of the points above.

Software and code

Policy information about availability of computer code

Data collection

1. For device, we have used AutoCAD 2015, Autodesk Inc., San Rafael, CA and we have used Solid-Works for 3D schematics.
2. For device fabrication, we have Aerosol Jet 3D printer (Model AJ-300, Optomec, Inc., Albuquerque, NM USA) with its software and x, y and z-software control panels. An in-built camera in this instrument with its measurement capability was used to obtain the pillar dimensions. Kewa Gadget, as an associated software having different control managers such as a motion manager 3.2.83, a process control 3.2.83, and vision manager 3.2.83 was used to control the 3D printing of nanoparticles.
3. We have utilized COMSOL Multiphysics® 5.5 software for device simulation,
4. For morphological and structural characterizations, we have used SEM (FEI Sirion SEM, Hillsboro, OR USA), XPS (Thetaprobe, ThermoFisher, Waltham, MA) and Raman instruments.
5. For electrochemical measurements, we have used VersaSTAT 3 Potentiostat Galvanostat, Princeton Applied Research, Oak Ridge, TN USA.
6. We have used OriginPro 2020 and GraphPad Prism 6 (One-way ANOVA) for graph plotting and analysis.

Data analysis

We have not used any algorithms for data representation. For data analysis/graphing, we have utilized commercial softwares such as
1. OriginPro (Version 2020)
2. GraphPad Prism 6 (One-way ANOVA)

For manuscripts utilizing custom algorithms or software that are central to the research but not yet described in published literature, software must be made available to editors and reviewers. We strongly encourage code deposition in a community repository (e.g. GitHub). See the Nature Portfolio guidelines for submitting code & software for further information.

March 2021

Data

Policy information about availability of data

All manuscripts must include a data availability statement. This statement should provide the following information, where applicable:

- Accession codes, unique identifiers, or web links for publicly available datasets
- A description of any restrictions on data availability
- For clinical datasets or third party data, please ensure that the statement adheres to our policy

Statement is included in the manuscript which is "All relevant data that support the findings of this study are presented in the manuscript and supplementary information file. Source data are available from the corresponding author upon reasonable request"

Field-specific reporting

Please select the one below that is the best fit for your research. If you are not sure, read the appropriate sections before making your selection.

[x] Life sciences   [ ] Behavioural & social sciences   [ ] Ecological, evolutionary & environmental sciences

For a reference copy of the document with all sections, see nature.com/documents/nr-reporting-summary-flat.pdf

Life sciences study design

All studies must disclose on these points even when the disclosure is negative.

Sample size

We would like to clarify that this is an engineering study for a biosensing technique rather than a life sciences study. The choice of sample sizes is given below.

a) For the repeatability study of the sensor (Figure 8c), 30 biologically independent experiments (n=30) across 10 sensors evenly split between 2D and 3D configuration were performed. Use of 15 experiments across 5 devices (randomly chosen with respect to the manufacturing sequence) gives a good indication of normality of data from design of experiments (Science advances, 2020, 1, 6(32), eabc4250; and Advanced Materials, 2021, 33, 2006647). Figure 8a and 8b have the same sample size justification. Once repeatability was established, storage stability of the device was studied with 15 biologically independent experiments across 12 days at an interval of three days were performed on a sensor to obtain Figure 8e and d. (Ref. Sensors and Actuators B: Chemical. 2006 Dec 7;119(2):419-24). Randomization with respect to time was not applicable because data was collected in a time sequence.
b) Sample size for spiked studies (Figure 6 and S10) was 60 biologically independent experiments (n=60) across two sensors evenly split between human plasma (Figure 6b) and artificial serum (Figure S10b). Two sensors were randomly chosen with respect to the manufacturing setup. One sensor was used to test 30 samples of spiked dopamine concentration (n=30; biological independent experiments) which were made in human plasma at five different concentration while other sensor was used to test 30 samples of spiked dopamine concentration (n=30; biological independent experiments) were made in artificial serum at five different concentration. Since the dopamine concentration was same across different dilution of human plasma and artificial serum, a total of 15 readings are obtained each with and without dopamine. This sample size is higher than 5 samples required to assess normality of the data. Figure 6a and 10Sa have the same sample size justification.
c) Sample size for selectivity studies (Figure 7) was 66 biological independent experiments (n=66) across two sensors evenly split between 2D (Figure 7b) and 3D sensor (Figure 7d) configuration. Two sensors were randomly chosen with respect to the manufacturing setup. The 2D sensor was used to test 33 samples that were made in different neurotransmitters with a fixed dopamine concentration (Figure 7b). Five different neurotransmitters were chosen in this experiment and a total of 33 experiments (15 experiments with other nuerotransmitters only and 18 experiments where a combination of other neurotransmitters and dopamine were used. This sample size was sufficient to establish the normality of the data and the selectivity of the device, which was the purpose of this study. The same argument holds for the 3D sensor configuration (sample size, n=33, Figure 7d). Figure 7a and 7c have the same sample size justification.
d) The aim for the experiment described in Figure 5 was to establish in-vitro analysis of the sensor in presence of rabbit serum and fetal bovine serum as well as demonstrate that the 3D sensor has a higher signal in all the cases. Sample size for this study was 42 (n=42, biological independent experiments) across two sensors evenly split between 2D (Figure 5b) and 3D (Figure 5d) configuration. Use of 21 experiments (n=21) across one 2D device (sensor chosen with respect to the manufacturing setup). A total of seven samples (21 readings) gives a good indication of normality of data from design of experiments (Figure 5b). Similarly, a same sample size (n=21) was used for 3D sensor configuration (Figure 5d). The two sensors used to collect data in Figure 5b and 5d were chosen randomly with respect to the manufacturing setup. The time sequence of experiments is as appears in the x-axis of the figures. In confirm that the experiments are not biased, pbs (i.e., baseline) was tested at the end of each sequence to confirm hat the signal goes back to the baseline. Figure 5a and 5c have the same sample size justification.
e) The aim of the titrate experiment described in Figure 4 was to carry out the titrate analysis and establish that the current is high for the 3D sensor compared to the 2D sensor. The sample size for this analysis was 63 biologically independent experiments (n=63) across three sensor configurations such as 2D (0×0), 3D (4×4) and 3D (10×10). For a 3D (10×10) sensor, a total sample size of eight (total readings from 24 biological independent experiments; n=24) was chosen for the serial titrate measurements (Figure 4f). Each concentration of dopamine was assessed with three repeat measurements (Nat. Comm.,12, Article number: 4039, 2021). The same sample size was used for the other two configurations as shown in Figures 4b and 4d. Since this is a titrate-type dose-dependent experiment, the dopamine concentration was increased serially (Biosens. Bioelectronic. 138, 2019, 111310). Figure 4a, 4b and 4c have the same sample size justification.

Data exclusions

No data was excluded from the analysis

Replication

For each experiments, we have repeated three replicate measurements as stated above Section of Sample Size.

Randomization	We have stated the randomization of the experiments in above Section of Sample size.
Blinding	This paper demonstrates a highly sensitive device for detection of dopamine. We use known concentration of dopamine and obtain a corresponding signal from sensor. There are no patient trials. As a result, blinding studies are not relevant for this paper.

Reporting for specific materials, systems and methods

We require information from authors about some types of materials, experimental systems and methods used in many studies. Here, indicate whether each material, system or method listed is relevant to your study. If you are not sure if a list item applies to your research, read the appropriate section before selecting a response.

Materials & experimental systems

n/a	Involved in the study
☒	☐ Antibodies
☒	☐ Eukaryotic cell lines
☒	☐ Palaeontology and archaeology
☒	☐ Animals and other organisms
☒	☐ Human research participants
☒	☐ Clinical data
☒	☐ Dual use research of concern

Methods

n/a	Involved in the study
☒	☐ ChIP-seq
☒	☐ Flow cytometry
☒	☐ MRI-based neuroimaging
